# Association of remnant cholesterol with kidney outcomes in adults with type 2 diabetes: a systematic review and meta-analysis

**DOI:** 10.3389/fendo.2026.1969361

**Published:** 2026-10-16

**Authors:** Jie Gao, Jiaqi Wang, Zhangli Wang

**Affiliations:** 1 The First Clinical Medical College, Yan'an Medical College, Yan'an University, Yan’an, Shaanxi, China; 2 Yan'an Medical College, Yan'an University, Yan'an, Shaanxi, China; 3 Department of Endocrinology, Affiliated Hospital of Yan'an University, Yan'an, Shaanxi, China; 4 Department of Nephrology, The Second Affiliated Hospital of Xi’an Jiaotong University, Xi’an, Shaanxi, China

**Keywords:** diabetic kidney disease, meta-analysis, remnant cholesterol, systematic review, type 2 diabetes mellitus

## Abstract

**Introduction:**

Remnant cholesterol (RC) reflects the cholesterol carried by triglyceride-rich lipoprotein remnants and may capture lipid-related kidney risk not fully represented by conventional lipid measures. We evaluated the associations between RC and kidney outcomes in adults with type 2 diabetes mellitus (T2DM).

**Methods:**

Following PRISMA 2020, PubMed/MEDLINE, Scopus, the Cochrane Library, Google Scholar, Wiley Online Library, and ScienceDirect were searched for longitudinal and cross-sectional observational studies evaluating circulating RC and kidney outcomes in adults with T2DM. Studies of mixed populations were eligible only when T2DM-specific data could be extracted. Adjusted effect estimates were pooled separately by study design and kidney outcome using restricted maximum likelihood random-effects models with Hartung–Knapp adjustment.

**Results:**

Seventeen articles contributed 18 independent estimates from 2,817,820 participants with T2DM. The exploratory all-study model showed an association between higher RC and adverse kidney outcomes (association ratio 1.50, 95% CI 1.32–1.72, *P* < 0.001; I^2^ = 87.6%). The longitudinal model, comprising 12 independent estimates from 11 articles, showed a similar association (HR 1.27, 95% CI 1.17–1.37, *P* < 0.001; I^2^ = 42.9%). Outcome-specific analyses showed an association with incident diabetic kidney disease (DKD; HR 1.27, 95% CI 1.15–1.41, *P* = 0.0029). The estimate for kidney failure/end-stage kidney disease was positive but not statistically significant (HR 1.56, 95% CI 0.82–2.97, *P* = 0.115). The association with DKD progression also did not reach statistical significance (HR 1.17, 95% CI 1.00–1.37, *P* = 0.052), while one study reported an association with incident albuminuria (HR 1.23, 95% CI 1.06–1.43, *P* = 0.007). Six cross-sectional studies showed an association between higher RC and prevalent or early DKD in adults with T2DM (OR 2.03, 95% CI 1.71–2.42, *P* < 0.001; I^2^ = 2.5%). The longitudinal association remained after restricting the analysis to estimates adjusted for blood pressure, glycemia, and adiposity (HR 1.28, 95% CI 1.21–1.37, *P* < 0.001). A smaller subset that included continuous estimates showed that a 0.1 mmol/L increase in RC was not associated with incident DKD (HR 1.02, 95% CI 0.98–1.07, *P* = 0.197).

**Conclusions:**

Higher RC was associated with incident and prevalent DKD and with adverse longitudinal kidney outcomes overall in adults with T2DM. RC may serve as a complementary kidney-risk marker, but the observational evidence does not establish causality or a clinical threshold.

**Systematic review registration**: https://www.crd.york.ac.uk/prospero/display_record.php?ID=CRD420261475085, identifier CRD420261475085.

## 1 Introduction

Diabetic kidney disease (DKD) is a major contributor to morbidity and mortality among people with diabetes mellitus (DM), particularly those with type 2 diabetes mellitus (T2DM), and is especially common among individuals with long-standing or poorly controlled hyperglycemia (1). According to the International Diabetes Federation, the global prevalence of diabetes continues to increase, and DKD affects approximately 20%–40% of people with diabetes (2, 3). Because its prevalence has not declined substantially in recent years, additional markers are needed to improve kidney-risk assessment in T2DM (4).

Remnant cholesterol (RC) refers to the cholesterol carried within triglyceride-rich lipoprotein (TRL) remnants. T2DM is commonly characterized by increased hepatic production of very-low-density lipoprotein (VLDL) and delayed clearance of TRLs; therefore, RC may reflect a component of the cholesterol burden that is not fully captured by conventional lipid measures (5). Higher RC has been associated with incident T2DM and cardiovascular events (6, 7). Evidence linking RC to kidney outcomes in adults with T2DM remains limited. Wu et al. reported associations of both baseline and cumulative RC levels with incident DKD (8). However, the available studies differed in RC exposure definitions, baseline kidney status, kidney outcome definitions, and covariate adjustment. An earlier systematic review reported an association between higher RC and CKD among individuals with diabetes, but combined cross-sectional and longitudinal studies and did not distinguish incident, prevalent, and progressive kidney outcomes (9). Separate analyses by study design and kidney outcome are therefore warranted to clarify the associations of RC with incident DKD, kidney function decline or progression, and kidney failure.

Accordingly, we conducted an updated systematic review and meta-analysis to evaluate the associations between RC and kidney outcomes in adults with T2DM. To address the interpretive challenges arising from previous syntheses that combined different study designs and outcomes, longitudinal and cross-sectional studies were analyzed separately, with outcome-specific evaluations of incident DKD, kidney function decline or progression, kidney failure/ESKD, incident albuminuria, and prevalent DKD. This study sought to clarify the associations between RC and distinct kidney outcomes and to provide an evidence base for further evaluation of its potential role in kidney risk among individuals with T2DM.

## 2 Materials and methods

This systematic review and meta-analysis were conducted and reported in accordance with the Preferred Reporting Items for Systematic Reviews and Meta-Analyses (PRISMA) 2020 statement (10). The protocol was registered in the International Prospective Register of Systematic Reviews (PROSPERO; registration number: CRD420261475085).

### 2.1 Search strategy

We searched PubMed/MEDLINE, Scopus, the Cochrane Library, Google Scholar, Wiley Online Library, and ScienceDirect from database inception to 16 September 2026. Google Scholar was used as a supplementary academic search engine, whereas Wiley Online Library and ScienceDirect were searched as publisher platforms. A total of 62 and 54 records were exported from PubMed and Scopus, respectively, and the first 50 pages of Google Scholar results were exported. An additional 130, 329, and 3 records were retrieved from Wiley Online Library, ScienceDirect, and the Cochrane Library, respectively. The complete search strategies, search fields, and numbers of records retrieved from each source are presented in **Table 1**.

**Table 1:** Identified keywords.

Database	Search strategy	Search fields	Numbers
PubMed	((remnant[Title/Abstract] AND cholesterol[Title/Abstract]) OR RLP-C[Title/Abstract]) AND ((type[Title/Abstract] AND 2[Title/Abstract] AND diabetes[Title/Abstract]) OR T2DM[Title/Abstract]) AND (kidney[Title/Abstract] OR renal[Title/Abstract] OR nephropathy[Title/Abstract] OR albuminuria[Title/Abstract] OR proteinuria[Title/Abstract] OR ACR[Title/Abstract] OR eGFR[Title/Abstract] OR creatinine[Title/Abstract] OR ESKD[Title/Abstract] OR ESRD[Title/Abstract])	Title/Abstract. From database inception. No date, language, or study-design restrictions.	62
Scopus	TITLE-ABS-KEY({remnant cholesterol} AND ({type 2 diabetes} OR T2DM) AND (kidney OR renal OR nephropathy OR albuminuria OR proteinuria OR ACR OR eGFR OR creatinine OR ESKD OR ESRD))	Title, abstract, and keywords. From database inception. No date, language, or study-design restrictions.	54
Google Scholar	(“remnant cholesterol” OR “remnant-C” OR “remnant lipoprotein cholesterol” OR “RLP-C”) (“type 2 diabetes” OR T2DM) (kidney OR renal OR nephropathy OR albuminuria OR proteinuria OR ACR OR eGFR OR CKD OR ESKD OR ESRD)	Supplementary scholarly search engine; sorted by relevance. No date or language restriction. The platform displayed 1,000 results, of which the first 500 were exported.	500
Cochrane Library	(“remnant cholesterol” OR “remnant-C” OR “remnant lipoprotein cholesterol” OR “RLP-C”) AND (“type 2 diabetes” OR T2DM) AND (kidney OR renal OR nephropathy OR albuminuria OR proteinuria OR ACR OR eGFR OR CKD OR ESKD OR ESRD)	Title, Abstract, and Keyword search. From database inception. No date, language, or study-design restrictions.	3
Wiley Online Library	(“remnant cholesterol” OR “remnant-C” OR “remnant lipoprotein cholesterol” OR “RLP-C”) AND (“type 2 diabetes” OR T2DM) AND (kidney OR renal OR nephropathy OR albuminuria OR proteinuria OR ACR OR eGFR OR CKD OR ESKD OR ESRD)	Publisher-platform search across all available fields. No date, language, or study-design restrictions.	130
ScienceDirect	(“remnant cholesterol” OR “remnant-C” OR “remnant lipoprotein cholesterol” OR “RLP-C”) AND (“type 2 diabetes” OR T2DM) AND (kidney OR renal OR nephropathy OR albuminuria OR proteinuria OR ACR OR eGFR OR CKD OR ESKD OR ESRD)	Publisher-platform search across all available fields. No date, language, or study-design restrictions.	329

### 2.2 Study selection

#### 2.2.1 Eligibility criteria

Studies were selected according to the PICOS framework and were eligible if they met the following criteria. Population (P): adults aged ≥18 years with T2DM; for studies involving mixed populations, data for participants with T2DM had to be separately extractable. Exposure (I): circulating RC measured directly or calculated from standard lipid parameters, including baseline or time-updated measurements during follow-up and expressed as categorical levels, per predefined absolute unit increment, or per 1-standard deviation increment. Comparator (C): In categorical analyses, the lower RC category defined by each study served as the reference group. Continuous analyses estimated the association with kidney outcomes per predefined unit or 1-standard deviation increase in RC. All comparisons were conducted within populations with T2DM. Outcomes (O): at least one kidney outcome, including incident or prevalent albuminuria, eGFR decline, DKD onset or progression, study-defined kidney progression or composite outcomes, kidney failure, initiation of kidney replacement therapy, or ESKD. Study design (S): longitudinal cohort, case-control, or cross-sectional studies. Studies were synthesized separately according to study design and kidney outcome.

#### 2.2.2 Exclusion criteria

1. Studies that included only individuals with type 1 diabetes or other non-T2DM populations, or from which T2DM-specific data could not be separately extracted.
2. Studies that did not evaluate RC as the exposure of interest or did not report an eligible kidney outcome.
3. Studies that did not report an eligible adjusted effect estimate or sufficient data from which one could be derived, as well as reviews, editorials, case reports, conference abstracts with insufficient data, *in vitro* studies, and animal studies.
4. For reports involving overlapping cohorts and the same kidney outcome, the most informative report was retained on the basis of sample size, completeness of follow-up, clarity of outcome definition, and extent of covariate adjustment.

#### 2.2.3 Screening process

Two review authors (JG and ZLW) independently screened the titles and abstracts and assessed the full texts of potentially eligible reports. Studies with uncertain eligibility during title and abstract screening underwent full-text review and, when necessary, adjudication by a third review author. Disagreements were resolved through discussion and consensus. Reasons for exclusion at the full-text stage were recorded using prespecified categories related to the population, exposure, outcomes, and study design.

### 2.3 Data extraction and outcome harmonization

Two review authors (JG and ZLW) independently reviewed the full texts of the included studies and extracted the data. Disagreements were resolved through discussion and consensus. Extracted information included study design, sample size, follow-up duration, body weight, BMI, blood pressure, HbA1c, fasting plasma glucose, RC levels, baseline kidney status, and kidney outcomes. Each data item was verified against the corresponding page, table, figure, or **Supplementary Material** of the original report, and missing information was recorded as “NA.”

For longitudinal cohort studies, adjusted HRs for incident DKD, DKD progression, kidney failure/ESKD, and incident albuminuria were analyzed separately by outcome. Longitudinal estimates were further stratified according to baseline kidney status as no baseline DKD, established DKD, or mixed status. Within the established-DKD group, DKD progression and kidney failure/ESKD were analyzed separately. Mutually exclusive baseline strata from the same study were combined using inverse-variance weighting before inclusion. These subgroup analyses were considered exploratory because few estimates were available. Outcomes reported by only one study were summarized descriptively. For cross-sectional studies, adjusted ORs for prevalent or early DKD were pooled. Because RC and kidney status were assessed concurrently, these associations were not interpreted as disease onset or progression.

Baseline eGFR and ACR were analyzed separately from the clinical kidney outcomes. When comparable data were available, eGFR values in the highest and lowest RC groups at study entry were pooled as mean differences. For ACR, ratios of medians between the highest and lowest RC groups were pooled on the logarithmic scale, with variances approximated from the interquartile ranges under a log-normal assumption. eGFR slopes and longitudinal changes in eGFR or ACR were not pooled because comparable change estimates and their variances were unavailable.

### 2.4 Study quality assessment

Two review authors (JG and ZLW) independently assessed study quality using the Newcastle–Ottawa Scale (NOS), with disagreements resolved through discussion and consensus. The standard cohort form was applied to longitudinal studies, whereas a prespecified adaptation of the case-control form was used for cross-sectional studies. The maximum score was nine stars, with 7–9, 4–6, and 0–3 stars classified as high, moderate, and low quality, respectively. The complete NOS forms and scoring criteria are provided in **Supplementary Table S1**.

### 2.5 Certainty of evidence

The certainty of evidence for each prespecified kidney outcome was assessed using the GRADE framework (11) and rated as high, moderate, low, or very low. Observational evidence was initially rated as low and adjusted according to standard GRADE criteria. Exploratory models combining different outcomes or RC exposure scales were not graded.

### 2.6 Statistical analysis

All analyses were performed using the metafor package (version 5.0-1) in R (version 4.6.1). An exploratory model first included all eligible studies. Longitudinal cohort and cross-sectional studies were then analyzed separately, and longitudinal estimates were further grouped by prespecified kidney outcome. Adjusted HRs, RRs, and ORs with 95% CIs were analyzed on the logarithmic scale and subsequently back-transformed; standard errors were calculated from the reported CIs when unavailable. For ratio measures, values greater than 1 indicated a positive association between higher RC and adverse kidney outcomes. All pooled analyses used random-effects models with restricted maximum likelihood estimation and Hartung–Knapp adjustment.

Baseline kidney markers were analyzed separately. eGFR was pooled as the mean difference between the highest and lowest RC groups. For ACR, ratios of medians were analyzed on the logarithmic scale, with variances estimated from the interquartile ranges under a log-normal assumption. These analyses represented exploratory baseline comparisons rather than longitudinal changes.

*Post hoc* continuous and dose–response analyses were conducted for incident DKD. Adjusted HRs originally reported per 1 mmol/L increase in RC were rescaled to a 0.1 mmol/L increment and pooled using a REML random-effects model with Hartung–Knapp inference. For categorical data, representative RC doses were assigned using reported means or medians; when unavailable, category midpoints were used, with open-ended categories estimated from the width of the adjacent interval. A three-knot restricted cubic spline model with study-clustered robust variance estimation was used to estimate HRs at RC concentrations of 0.60, 0.80, and 1.00 mmol/L, using 0.40 mmol/L as the reference.

Cochran’s Q was used to test for between-study heterogeneity, I² quantified its relative magnitude. A 95% prediction interval was calculated where estimable. All statistical tests were two-sided, with P<0.05 considered statistically significant.

#### 2.6.1 Sensitivity analysis

To evaluate the influence of individual estimates, leave-one-out sensitivity analyses were performed separately for the longitudinal cohort and cross-sectional models. Each independent estimate was omitted in turn, and the model was refitted using the same REML random-effects method with Hartung–Knapp adjustment. Changes in the pooled estimate, 95% CI, and I² were examined. Results were considered robust when the direction, magnitude, and statistical interpretation of the association remained materially unchanged.

## 3 Results

### 3.1 Study selection and characteristics

The PRISMA 2020 flow diagram summarizes the study-selection process (**Figure 1**). The searches identified 1,078 records across PubMed/MEDLINE, Scopus, Google Scholar, the Cochrane Library, Wiley Online Library, and ScienceDirect. After removing 166 duplicates, 912 records underwent title and abstract screening, of which 889 were excluded for not meeting the prespecified PICOS criteria. All 23 remaining reports were assessed in full text, and six were excluded: one evaluated an RC-related ratio rather than RC itself; one did not include RC as an independent exposure in the kidney-outcome model, one did not provide separately extractable T2DM data, two lacked eligible adjusted effect estimates, and one was an overlapping preprint. Seventeen articles were ultimately included, comprising 11 longitudinal cohort and six cross-sectional studies. Chen et al. reported two independent cohorts, the UK Biobank and Chinese Diabetes Registry, resulting in 18 independent estimates.

**Figure 1:**
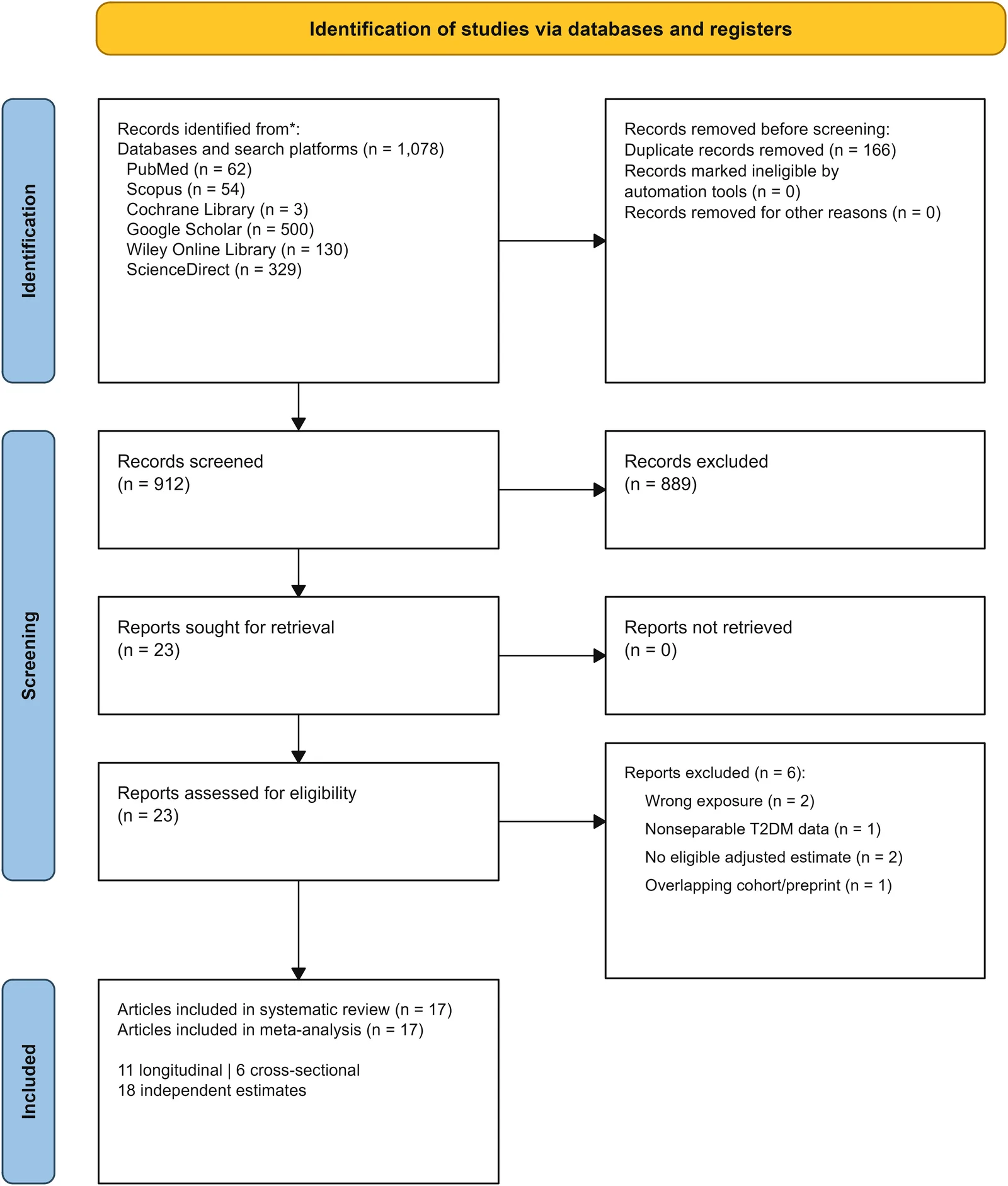
PRISMA 2020 flow diagram of study selection.

The 17 articles collectively reported 2,817,820 participants from China, Japan, South Korea, the United Kingdom, and the United States. The study populations included participants without kidney disease at baseline, general T2DM populations, and cohorts with established DKD (**Table 2**) (6, 8, 12–26). RC was generally calculated as total cholesterol minus high-density lipoprotein cholesterol and low-density lipoprotein cholesterol; two studies used Martin–Hopkins-based calculations. Most studies assessed baseline RC using tertiles, quartiles, study-defined thresholds, predefined unit increments, or 1-standard deviation increments, whereas one study evaluated cumulative RC using repeated measurements during follow-up. Longitudinal outcomes included incident DKD, incident albuminuria, kidney function or eGFR decline, DKD progression, kidney failure, and ESKD. Cross-sectional studies primarily assessed prevalent or early DKD. Several studies also reported baseline eGFR, ACR, or 24-hour urinary protein (**Table 2**).

**Table 2:** Characteristics of included studies.

Study	Country; design	Diabetes; population	Sample size	Age; male	DM duration; follow-up	BMI	SBP/DBP	HbA1c; FPG	TC	TG	HDL-C	LDL-C	RC	Proteinuria/ACR	eGFR
Shang 2026 (26)	China; Retrospective cohort	T2DM; Hospitalized adults with established DKD	472; kidney failure 435	63.00 [55.00-75.00]; 69.3% male	DM: NR; FU: 26.4 [16.5-35.0] months	NR	NR	HbA1c: 8.30 [7.08-9.72]; FPG: NR	4.12 [3.32-5.10] mmol/L	1.67 [1.17-2.63] mmol/L	1.17 [1.02-1.37] mmol/L	2.11 [1.61-2.78] mmol/L	0.76 [0.53-1.05] mmol/L	Semiquantitative dipstick proteinuria; ACR NR	59.42 [43.71-86.80]
Li 2026 (25)	China; Multicenter cross-sectional	T2DM; Hospitalized adults with T2DM	1893	non-DKD/DKD: 61 [51-69]/65 [57-72]; 52.61%/53.89% male	DM: NR; FU: NA	24.10 [21.92-26.75]/24.51 [22.03-26.73]	NR	HbA1c: 9.6 [7.4-11.8]/9.0 [7.3-11.3]; FPG: 7.35 [5.37-10.19]/6.74 [5.10-9.88]	4.58 [3.84-5.36]/4.56 [3.78-5.46] mmol/L	1.40 [1.02-2.00]/1.47 [1.02-2.13] mmol/L	1.02 [0.86-1.19]/0.99 [0.84-1.16] mmol/L	3.00 [2.25-3.72]/2.91 [2.18-3.75] mmol/L	0.46 [0.31-0.67]/0.51 [0.36-0.75] mmol/L	ACR 16.90 [6.95-76.50]/65.40 [20.35-169.40] mg/g	93.26 [76.99-110.31]/78.49 [51.73-100.91]
Dai 2025 (15)	China; Retrospective cohort	T2DM; T2DM without baseline DKD	2122	57.47 ± 11.19; 62.44% male	DM: 7.00 [3.00-10.00]; FU: 4.22	24.65 ± 4.35	133.91 ± 18.05/78.87 ± 11.24	HbA1c: 8.34 ± 2.24; FPG: 7.72 ± 2.80	4.70 ± 1.12 mmol/L	1.47 [1.03-2.20] mmol/L	1.12 ± 0.30 mmol/L	2.88 ± 0.82 mmol/L	0.64 [0.47-0.83] mmol/L	ACR 10.10 [5.20-16.13] mg/g	78.44 ± 8.95
Mori 2026	Japan; Retrospective cohort	T2DM; Adults with T2DM	5214	61 ± 12; 63.7% male	DM: 13 ± 9; FU: 8.8 [4.2-14.9]	24.7 ± 4.0	138 ± 21/77 ± 12	HbA1c: 7.7 ± 1.3; FPG: NR	199 ± 37 mg/dL	128 [85-187] mg/dL	54 ± 15 mg/dL	117 ± 31 mg/dL	24.0 [17.0-33.0] mg/dL	ACR 27.1 [7.5-65.1] mg/g	70.9 ± 22.7
Chen 2025-1 (14)	United Kingdom; Prospective cohort	T2DM; UK Biobank T2DM cohort	23324; longitudinal 20892	59.56 ± 7.20; 61.25% male	DM: NR; FU: 13.72	31.27 ± 5.89	143.20 ± 18.64/81.45 ± 10.40	HbA1c: 6.93 ± 1.28; FPG: 7.47 ± 3.39	4.40 [3.80-5.13] mmol/L	1.82 [1.26-2.59] mmol/L	1.15 [0.98-1.38] mmol/L	2.61 [2.18-3.15] mmol/L	0.56 [0.41-0.73] mmol/L	ACR 14.21 [7.71-33.93] mg/g	88.65 ± 16.73
Chen 2025-2 (14)	China; Prospective registry cohort	T2DM; Chongqing Diabetes Registry	3059; longitudinal 1130	58.12 ± 11.23; 59.43% male	DM: NR; FU: 1.92	24.76 ± 5.92	134.48 ± 19.35/78.81 ± 12.02	HbA1c: 9.12 ± 2.90; FPG: 8.90 ± 3.30	4.27 [3.58-5.07] mmol/L	1.51 [1.04-2.38] mmol/L	1.08 [0.89-1.34] mmol/L	2.51 [1.87-3.19] mmol/L	0.44 [0.25-0.74] mmol/L	ACR 15.9 [6.2-66.4] mg/g	91.61 ± 24.05
Roh 2025 (18)	South Korea; Nationwide retrospective cohort	T2DM; Korean NHIS adults with T2DM	2537149	Q1/Q4: 58.56 ± 12.48/54.57 ± 12.24; 55.60%/67.59% male	DM: NR; FU: 10.3 [10.1-10.6]	23.99 ± 3.35/25.89 ± 3.28	126.47 ± 15.68/76.88 ± 10.04; 131.17 ± 15.86/81.20 ± 10.39	HbA1c: NR; FPG: Q1/Q4: 136.99 ± 42.48/155.56 ± 52.57 mg/dL	Q1/Q4: 174.14 ± 35.69/223.43 ± 42.45 mg/dL	Q1/Q4: 77 [63-88]/278 [233-350] mg/dL	Q1/Q4: 56.34 ± 14.66/46.77 ± 11.91 mg/dL	Q1/Q4: 101.38 ± 32.02/130.70 ± 37.29 mg/dL	Q1/Q4: 16.41 ± 2.29/45.96 ± 15.47 mg/dL	NR	CKD-EPI Q1/Q4: 83.11 ± 20.10/83.48 ± 20.67
Da 2025 (16)	China; Longitudinal hospital cohort	T2DM; Hospitalized adults with T2DM	1219	59.7 ± 14.6; 58.6% male	DM: NR; FU: 4.5	NR	NR	HbA1c: NR; FPG: 12.8 [8.4-17.6]	4.37 [3.66-5.14] mmol/L	1.61 [1.06-2.42] mmol/L	0.96 [0.79-1.13] mmol/L	2.39 ± 0.87 mmol/L	0.88 [0.64-1.25] mmol/L	ACR 28 [9-154] mg/g	84.8 ± 30.6
Yu 2025 (17)	United States and Canada; *Post hoc* longitudinal cohort	T2DM; ACCORD participants with T2DM	10196	62.77 ± 6.63; 61.5% male	DM: 10.80 ± 7.59; FU: 7.0	32.23 ± 5.40	136.35 ± 17.11/74.88 ± 10.66	HbA1c: 8.30 ± 1.06; FPG: 175.20 ± 56.18 mg/dL	183.31 ± 41.85 mg/dL	155 [106-229] mg/dL	41.87 ± 11.62 mg/dL	104.90 ± 33.91 mg/dL	31.00 [21.00-46.00] mg/dL	ACR 14.00 [7.00-47.00] mg/g	91.05 ± 27.15
Zhao 2024 (13)	China; Longitudinal biopsy cohort	T2DM; T2DM with biopsy-confirmed diabetic nephropathy	334	51.1 ± 9.3; 70.0% male	DM: 96 [36-132] months; FU: 27 [17-43] months	25.6 ± 3.7	NR	HbA1c: 7.6 ± 1.9; FPG: 8.4 ± 4.3	5.0 [4.2-6.1] mmol/L	1.8 [1.3-2.4] mmol/L	1.2 [1.0-1.5] mmol/L	2.9 [2.2-3.7] mmol/L	0.68 [0.46-1.02] mmol/L	24-h protein 3.8 [1.8-7.0] g/day	60.7 [44.1-92.6]
LiQ 2024 (12)	China; Cross-sectional plus longitudinal cohort	T2DM; T2DM-related CKD	8678; longitudinal 6165	59.56 ± 9.97; 57.3% male	DM: NR; FU: 3.0	26.88 [24.62-29.40]	140.34 ± 18.70/NR	HbA1c: 8.41 ± 1.82; FPG: 9.16 [7.65-11.58]	5.23 ± 1.22 mmol/L	1.62 [1.16-2.40] mmol/L	1.20 [1.04-1.40] mmol/L	3.34 ± 0.99 mmol/L	0.55 [0.39-0.80] mmol/L	ACR 162.69 [62.74-386.02] mg/g; 24-h protein 0.30 [0.14-1.03] g/day	97.52 ± 30.41
Jang 2024 (6)	South Korea; Nationwide retrospective cohort	T2DM; Newly diagnosed T2DM without baseline CKD	212836	T1/T3: 50.2 ± 12.5/48.0 ± 10.9; 62.1%/83.7% male	DM: Newly diagnosed; FU: 5.23	23.4 ± 3.3/26.0 ± 3.4	124.2 ± 14.9/77.2 ± 9.9; 129.5 ± 14.7/81.2 ± 10.1	HbA1c: NR; FPG: T1/T3: 125.9 ± 31.7/139.5 ± 34.5 mg/dL	T1/T3: 188.6 ± 35.3/213.6 ± 39.1 mg/dL	T1/T3: 79.2 ± 24.7/252.2 ± 60.6 mg/dL	T1/T3: 58.9 ± 14.3/47.9 ± 11.5 mg/dL	T1/T3: 114.3 ± 32.9/115.0 ± 37.7 mg/dL	T1/T3: 15.4 ± 4.0/32.5 ± 28.2 mg/dL	NR	T1/T3: 93.2 ± 15.3/92.7 ± 15.2
Zhu 2024 (23)	United States; Cross-sectional	T2DM; Separately extractable NHANES T2DM subgroup	1473	NR; NR male	DM: NR; FU: NA	NR	NR	HbA1c: NR; FPG: NR	NR	NR	NR	NR	NR	NR	NR
Yuan 2023 (21)	China; Cross-sectional	T2DM; Hospitalized adults with T2DM	3383	54.2 ± 13.6; 61.4% male	DM: 8.0 [2.0-15.0]; FU: NA	26.3 ± 4.0	133.8 ± 18.1/79.4 ± 11.3	HbA1c: 9.6 ± 2.2; FPG: 143.9 ± 55.1 mg/dL	181.1 ± 43.5 mg/dL	139.9 [100.9-204.6] mg/dL	39.2 ± 10.6 mg/dL	109.8 ± 33.2 mg/dL	25.9 [18.6-36.7] mg/dL	CKD defined jointly by ACR/eGFR; overall ACR NR	NR
Wu 2022 (8)	China; Longitudinal cohort	T2DM; T2DM without baseline nephropathy or retinopathy	4237	55.80 ± 12.79; 76.8% male	DM: NR; FU: 5.0	26.37 [24.35-28.62]	NR	HbA1c: 6.32 [6.20-6.92]; FPG: 7.35 [6.53-8.63]	185.57 [158.51-211.86] mg/dL	139.10 [94.80-212.64] mg/dL	44.85 [38.62-52.96] mg/dL	111.34 [87.76-134.92] mg/dL	24.91 [18.99-33.84] mg/dL	NR	NR
Xu 2023	China; Cross-sectional	T2DM; Elderly hospitalized adults with T2DM	843	non-DKD/DKD: 71.37 ± 5.35/72.91 ± 6.18; 53.7%/55.4% male	DM: 13.0 [8.0-19.0]/16.0 [10.0-20.0]; FU: NA	23.07 ± 3.21/24.12 ± 3.42	136.43 ± 19.13/77.71 ± 13.65; 143.86 ± 23.08/76.84 ± 10.32	HbA1c: 8.65 [7.02-9.51]/8.60 [7.27-9.50]; FPG: 6.81 [5.76-8.20]/6.90 [5.78-8.30]	4.13 [3.49-4.79]/4.26 [3.50-4.91] mmol/L	1.30 [0.90-1.81]/1.46 [0.99-2.10] mmol/L	1.07 [0.91-1.29]/1.04 [0.88-1.21] mmol/L	2.56 [1.81-3.15]/2.56 [1.99-3.26] mmol/L	0.44 [0.37-0.55]/0.50 [0.39-0.63] mmol/L	ACR 7.00 [3.80-11.00]/133.30 [44.00-650.80] mg/g	90.98 [79.09-108.91]/64.56 [45.61-84.98]
Ma 2022	China; Cross-sectional prediction study	T2DM; Hospitalized adults with T2DM	978	training/test: 57 [47-67]/58 [49-66]; 63.5%/64.2% male	DM: 7 [1-13]/6 [1-10]; FU: NA	25.09 [22.76-27.59]/24.69 [23.10-27.68]	130 [120-140]/80 [77-90]; 130 [120-140]/80 [79-90]	HbA1c: 8.60 [7.10-10.10]/8.10 [7.00-9.90]; FPG: 8.24 [6.43-10.53]/8.03 [6.18-10.63]	4.60 [3.86-5.34]/4.64 [3.86-5.34] mmol/L	1.34 [0.95-2.03]/1.45 [1.03-2.26] mmol/L	1.03 [0.87-1.23]/1.02 [0.86-1.23] mmol/L	2.74 [2.06-3.42]/2.79 [2.14-3.40] mmol/L	0.65 [0.45-0.94]/0.66 [0.46-0.96] mmol/L	ACR measured; baseline summary NR	107.10 [96.60-120.00]/108.15 [99.33-120.00]
Guo 2024	China; Cross-sectional	T2DM; Consecutive hospitalized adults with T2DM	410	non-DKD/early DKD/clinical DKD: 56.18 ± 12.43/56.96 ± 14.02/57.83 ± 13.23; 163/106, 51/29, 41/20 (overall 62.2% male) male	DM: 5 [1-10]/10 [2-16.75]/10 [5-15.5] years; FU: NA	24.69 ± 4.28/25.60 ± 3.63/25.35 ± 3.58	129.84 ± 17.93/79.14 ± 11.30; 138.18 ± 19.58/81.44 ± 12.83; 143.57 ± 24.02/83.70 ± 12.59 mmHg	HbA1c: NR; FPG: 6.50 [5.28-8.30]/6.86 [5.54-8.85]/7.45 [6.21-8.53] mmol/L	4.52 [3.91-5.18]/4.96 [4.12-5.94]/5.11 [4.34-6.72] mmol/L	1.41 [1.00-1.97]/1.73 [1.24-2.47]/1.78 [1.20-2.64] mmol/L	1.22 ± 0.30/1.20 ± 0.31/1.21 ± 0.36 mmol/L	2.62 [2.12-3.14]/2.82 [2.31-3.52]/2.95 [2.42-4.00] mmol/L	0.65 [0.45-0.91]/0.93 [0.55-1.15]/0.92 [0.60-1.44] mmol/L	ACR 6 [4-11]/75 [49.5-146.5]/1177 [625-2848] mg/g	NR

BMI, body mass index; CKD, chronic kidney disease; DKD, diabetic kidney disease; DBP, diastolic blood pressure; DM, diabetes mellitus; eGFR, estimated glomerular filtration rate; ESKD, end-stage kidney disease; FPG, fasting plasma glucose; FU, follow-up; HDL-C, high-density lipoprotein cholesterol; LDL-C, low-density lipoprotein cholesterol; RC, remnant cholesterol; SBP, systolic blood pressure; TC, total cholesterol; TG, triglycerides; T2DM, type 2 diabetes mellitus; ACR, urinary albumin-to-creatinine ratio.

BMI was reported in 14 articles, including 10 using means and standard deviations and four using medians and interquartile ranges. Blood pressure was reported in 11 articles, of which 10 used means and standard deviations. HbA1c was reported in 12 articles, including seven using means and standard deviations. Fasting plasma glucose was reported in 14 articles, with seven using each statistical format. Eight articles reported numerical diabetes-duration data, and one included only patients with newly diagnosed T2DM. These characteristics are presented in **Table 2**, and adjustment for BMI or obesity, blood pressure, glycemia, and diabetes duration is summarized in **Supplementary Table S2**.

#### 3.1.1 Study quality and certainty of evidence

Thirteen articles were rated as high quality and four as moderate quality; none was rated as low quality. All 11 longitudinal articles received 7–9 NOS stars. Among the six cross-sectional articles assessed using the adapted case-control NOS, two were rated as high quality and four as moderate quality (**Supplementary Table S1**; **Supplementary Figure S1**).

GRADE certainty was low for incident DKD and very low for kidney function decline or progression, kidney failure/ESKD, incident albuminuria, and prevalent DKD. The all-study and longitudinal overview models were not assigned a single GRADE rating because they combined different kidney outcomes and/or RC exposure contrasts (**Supplementary Table S3**).

### 3.2 RC and kidney outcomes in T2DM

#### 3.2.1 Overall model

The exploratory overall model included 18 independent estimates from 17 articles. Higher RC was associated with adverse kidney outcomes (association ratio 1.50, 95% CI 1.32–1.72, *P* < 0.001; I^2^ = 87.6%, Q = 67.63, *P* for heterogeneity <0.001; **Figure 2**; **Table 3**). The 95% prediction interval crossed the null value (0.95–2.37). Because this model combined HRs and ORs, longitudinal and cross-sectional designs, different kidney outcomes, and different RC contrasts, it was interpreted as an exploratory overall association rather than a common causal or clinical effect.

**Figure 2:**
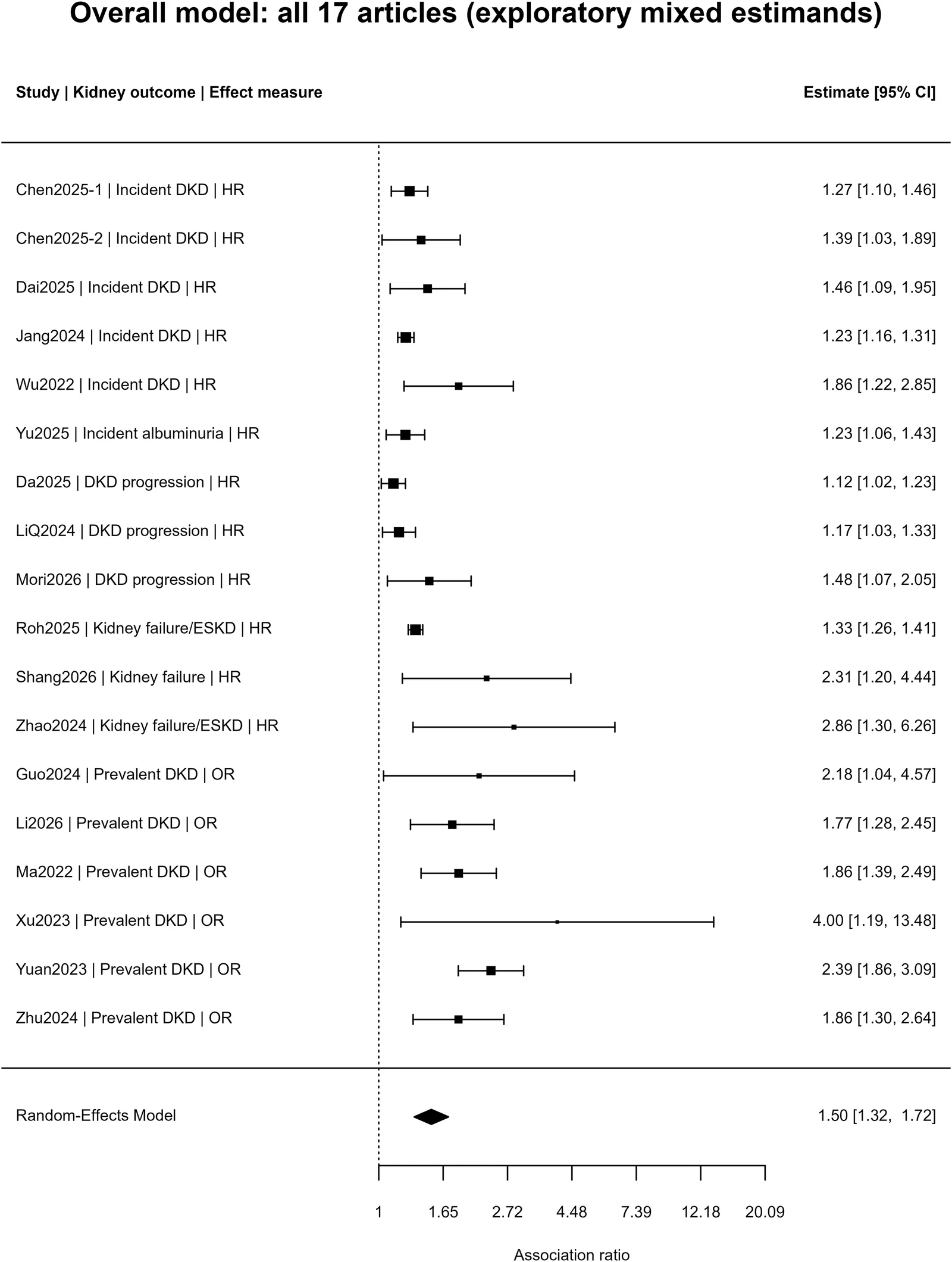
Exploratory overall association between remnant cholesterol and kidney outcomes in adults with type 2 diabetes. Squares and horizontal lines indicate study-specific estimates and 95% confidence intervals, respectively; the diamond indicates the pooled estimate from the random-effects model. The dashed vertical line indicates the null value of 1. CI, confidence interval; DKD, diabetic kidney disease; ESKD, end-stage kidney disease; HR, hazard ratio; OR, odds ratio.

**Table 3:** Summary of meta-analysis results.

Analysis	Role	Effect	Articles/estimates	Pooled effect [95% CI]	*P* value	I^2^, %	tau^2^	Q *P* value	Prediction interval
All 17 articles	Overall exploratory	Mixed HR/OR	17/18	1.50 [1.32, 1.72]	<0.001	87.6	0.04282	<0.001	0.95-2.37
Longitudinal cohort studies	Longitudinal overview	HR	11/12	1.27 [1.17, 1.37]	<0.001	42.9	0.00321	0.011	1.09-1.47
cross-sectional studies	Cross-sectional overview	OR	6/6	2.03 [1.71, 2.42]	<0.001	2.5	0.00093	0.536	1.68-2.46
Incident DKD	Primary	HR	4/5	1.27 [1.15, 1.41]	0.003	7.4	0.00064	0.281	1.12-1.44
Incident DKD restricted to NOS 8-9	Primary sensitivity	HR	3/4	1.25 [1.17, 1.34]	0.002	0.0	0.00000	0.619	1.17-1.34
Kidney function decline/progression	Secondary	HR	3/3	1.17 [1.00, 1.37]	0.052	0.1	0.00000	0.340	1.00-1.37
Kidney failure/ESKD	Secondary	HR	4/4	1.56 [0.82, 2.97]	0.115	69.5	0.08858	0.067	0.50-4.90
Incident albuminuria	Secondary	HR	1/1	1.23 [1.06, 1.43]	0.007	NA	NA	NA	NA

Random-effects models used restricted maximum likelihood estimation with Hartung-Knapp inference. The all-study model combines HRs and ORs, longitudinal and cross-sectional designs, kidney-outcome domains, and remnant-cholesterol contrasts and is therefore an exploratory association ratio rather than a common causal effect. Incident albuminuria was reported by one study and was not pooled. CI, confidence interval; DKD, diabetic kidney disease; ESKD, end-stage kidney disease; HR, hazard ratio; I2, heterogeneity statistic; NOS, Newcastle-Ottawa Scale; OR, odds ratio.

#### 3.2.2 Longitudinal cohort model

The longitudinal model included 12 independent cohort estimates from 11 articles. Higher RC was associated with adverse kidney outcomes (HR 1.27, 95% CI 1.17–1.37, *P* < 0.001; I²=42.9%; Q = 24.50, *P* for heterogeneity=0.011; **Figure 3**; **Table 3**). In leave-one-out analyses, pooled HRs ranged from 1.24 to 1.29, all 95% CIs remained above 1, and all *P* values were <0.001; I^2^ ranged from 17.1% to 52.1%. The findings were therefore not materially influenced by any single estimate (**Figure 3**).

**Figure 3:**
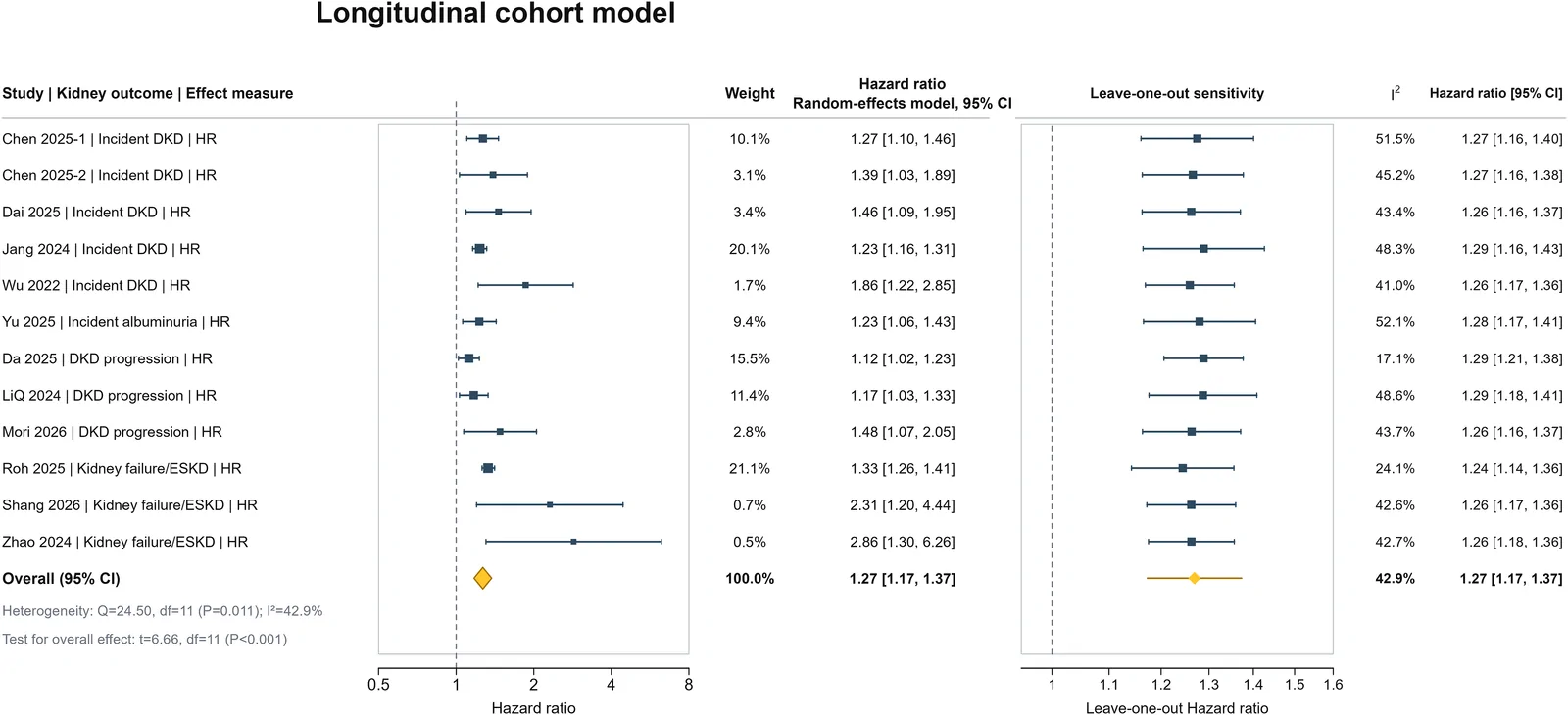
Longitudinal association between remnant cholesterol and kidney outcomes in adults with type 2 diabetes. The left panel presents the random-effects model based on 12 independent cohort estimates. The right panel presents leave-one-out sensitivity analyses in which each corresponding estimate was omitted in turn and the model was refitted using the same analytical method. Squares and horizontal lines represent individual estimates and 95% confidence intervals; diamonds represent pooled estimates. The dashed vertical line indicates the null value of 1. CI, confidence interval; DKD, diabetic kidney disease; ESKD, end-stage kidney disease; HR, hazard ratio; RC, remnant cholesterol.

#### 3.2.3 Outcome-specific longitudinal analyses

Five independent cohorts from four articles evaluated incident DKD. Higher RC was associated with an increased risk of incident DKD (HR 1.27, 95% CI 1.15–1.41, *P* = 0.0029; I^2^ = 7.4%; Q = 5.07, *P* for heterogeneity=0.281; **Figure 4**; **Table 3**). Restriction to four estimates from studies scoring 8–9 NOS stars produced a similar result (HR 1.25, 95% CI 1.17–1.34, *P* = 0.0019; I^2^ = 0%; Q = 1.78, *P* for heterogeneity=0.619; **Figure 4**; **Table 3**).

**Figure 4:**
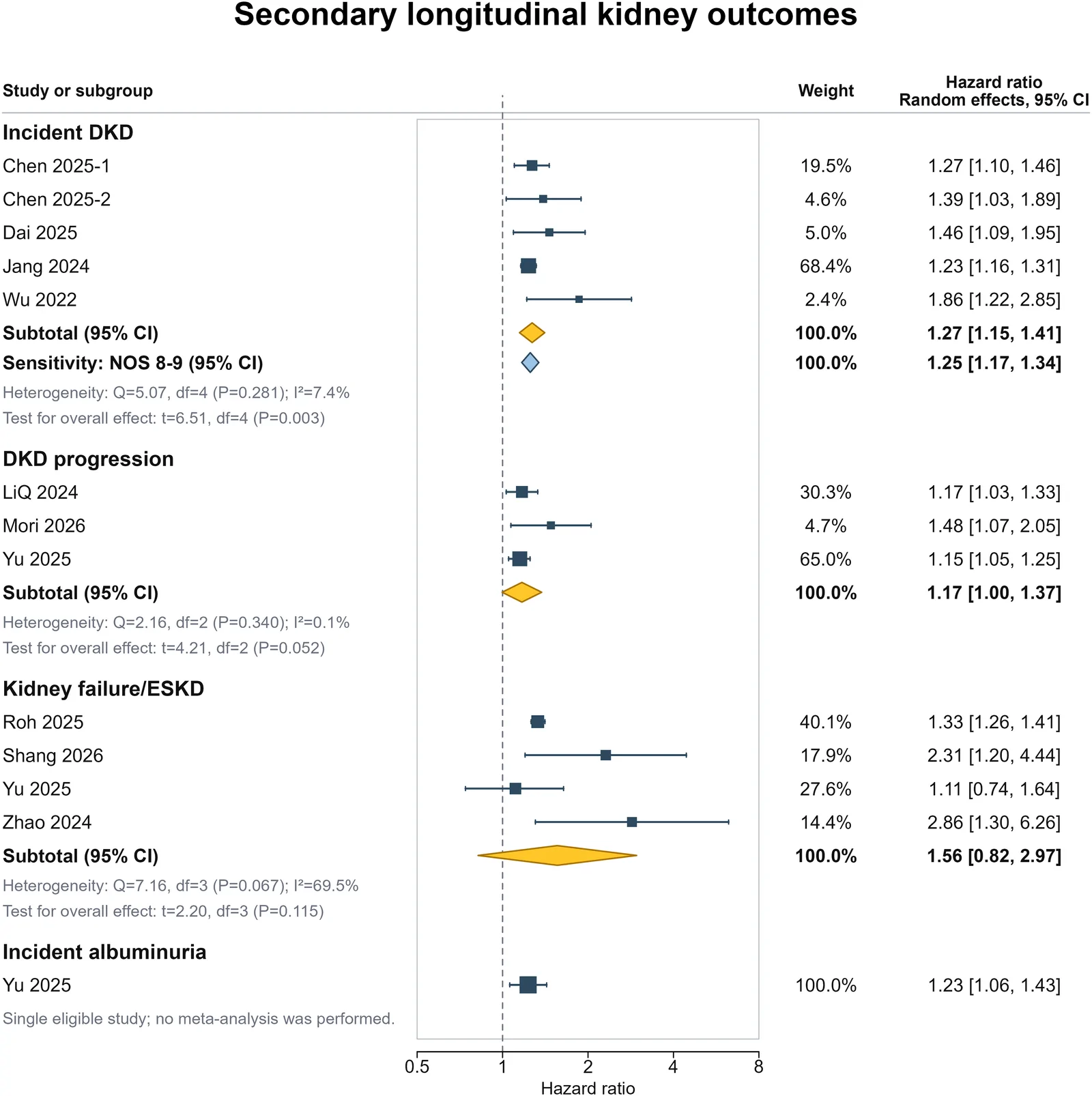
Outcome-specific longitudinal associations between remnant cholesterol and kidney outcomes in adults with type 2 diabetes. Separate random-effects analyses are presented for incident diabetic kidney disease, diabetic kidney disease progression, kidney failure or end-stage kidney disease, and incident albuminuria. For incident diabetic kidney disease, the yellow diamond represents the analysis of all eligible estimates and the blue diamond represents the sensitivity analysis restricted to studies scoring 8–9 stars on the Newcastle–Ottawa Scale. Incident albuminuria was reported by one eligible study and was therefore not pooled. Squares and horizontal lines indicate study-specific hazard ratios and 95% confidence intervals; diamonds indicate pooled estimates. CI, confidence interval; DKD, diabetic kidney disease; ESKD, end-stage kidney disease; HR, hazard ratio; NOS, Newcastle–Ottawa Scale; RC, remnant cholesterol.

Three studies evaluated DKD progression. Higher RC showed a positive association with DKD progression, although the result was not statistically significant (HR 1.17, 95% CI 1.00–1.37, *P* = 0.052; I^2^ = 0.1%; Q = 2.16, *P* for heterogeneity=0.340). Four studies reported kidney failure/ESKD, the pooled estimate was positive but did not reach statistical significance (HR 1.56, 95% CI 0.82–2.97, *P* = 0.115; I^2^ = 69.5%; Q = 7.16, *P* for heterogeneity=0.067). One study reported an association between higher RC and incident albuminuria (HR 1.23, 95% CI 1.06–1.43, *P* = 0.0067); therefore, this outcome was not pooled (**Figure 4**; **Table 3**).

Subgroup analyses stratified by baseline kidney status are shown in **Figure 5**. Among cohorts without baseline DKD, six estimates were available. Among cohorts without baseline DKD, higher RC was associated with incident DKD or subsequent kidney deterioration (HR 1.31, 95% CI 1.15–1.50, *P* = 0.003; I^2^ = 26.1%). Among cohorts with established DKD, higher RC was positively associated with DKD progression (HR 1.17, 95% CI 1.04–1.33, *P* = 0.039; I^2^ = 0%). For kidney failure/ESKD, the pooled estimate was positive but did not reach statistical significance (HR 2.52, 95% CI 0.67–9.52, *P* = 0.072; I^2^ = 0%). Among cohorts with mixed baseline kidney status, higher RC was positively associated with adverse kidney outcomes (HR 1.22, 95% CI 1.03–1.45, *P* = 0.033; I^2^ = 76.8%).

**Figure 5:**
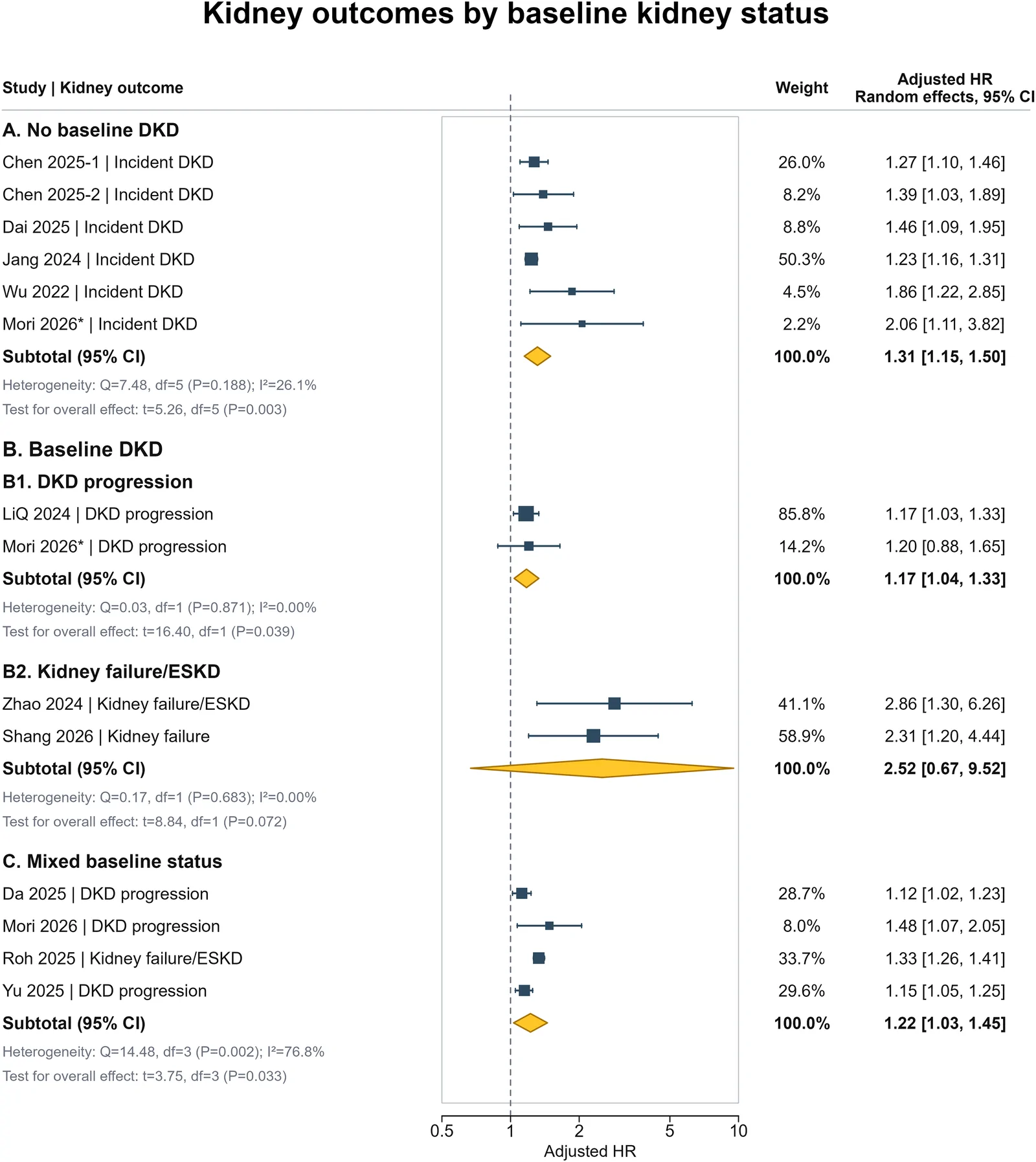
Kidney outcomes according to baseline kidney status. **(A)** Incident DKD among participants without baseline DKD. **(B)** Outcomes among participants with established DKD: DKD progression (B1) and kidney failure/ESKD (B2). **(C)** Kidney outcomes in cohorts with mixed baseline kidney status. *For Mori et al., the estimate in panel A was restricted to participants with an eGFR ≥60 mL/min/1.73 m² and an ACR <30 mg/g at baseline; incident DKD was operationalized as a sustained ≥40% decline in eGFR for ≥90 days. In panel B1, three mutually exclusive baseline DKD strata were combined using inverse-variance weighting before inclusion. Squares and horizontal lines indicate study-specific hazard ratios and 95% confidence intervals; diamonds indicate pooled estimates. CI, confidence interval; DKD, diabetic kidney disease; ESKD, end-stage kidney disease; HR, hazard ratio; RC, remnant cholesterol.

#### 3.2.4 Continuous associations

Direct continuous estimates from two articles comprising three cohorts showed that the pooled HR for incident DKD was 1.02 per 0.1 mmol/L increase in RC (95% CI 0.98–1.07, *P* = 0.197; I²=81.4%; Q = 9.85, *P* for heterogeneity=0.007; **Figure 6A**). The association was not statistically significant.

**Figure 6:**
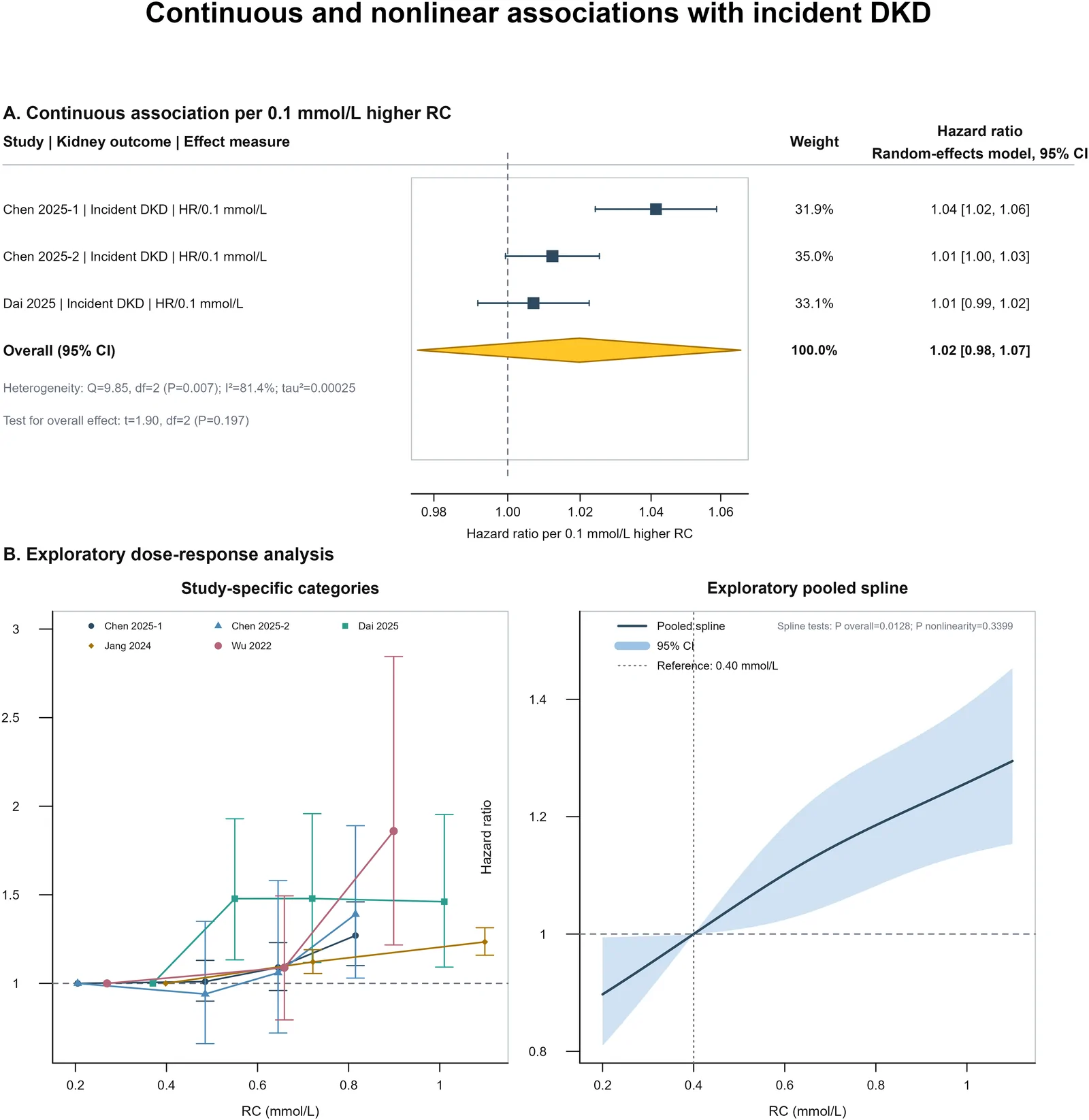
Continuous and exploratory dose–response associations between remnant cholesterol and incident diabetic kidney disease. **(A)** Forest plot of direct continuous estimates from two articles comprising three independent cohorts, standardized to a 0.1 mmol/L increase in remnant cholesterol. **(B)** Exploratory dose–response analysis based on five cohorts from four articles. The left plot shows study-specific categorical estimates, with representative doses derived from reported means, medians, or category boundaries. The right plot shows the pooled three-knot restricted cubic spline and its 95% confidence interval, using an RC concentration of 0.40 mmol/L as the reference. CI, confidence interval; DKD, diabetic kidney disease; HR, hazard ratio; RC, remnant cholesterol.

In the exploratory dose–response analysis, using 0.40 mmol/L as the reference, the predicted HRs were 1.10 (95% CI 1.02–1.19), 1.19 (95% CI 1.08–1.30), and 1.26 (95% CI 1.14–1.39) at RC concentrations of 0.60, 0.80, and 1.00 mmol/L, respectively. The overall dose–response association was statistically significant (P = 0.0128), whereas the test for nonlinearity was not (P = 0.340). Thus, no distinct nonlinear pattern or common RC threshold was identified (**Figure 6B**; **Supplementary Table S4**).

#### 3.2.5 Cross-sectional model

Six cross-sectional studies were included (**Figure 7**; **Table 3**). Higher RC was associated with prevalent or early DKD (OR 2.03, 95% CI 1.71–2.42, *P* < 0.001; I²=2.5%; Q = 4.10, *P* for heterogeneity=0.536). This finding represents a prevalence association and does not establish temporal order. In leave-one-out analyses, pooled ORs ranged from 1.88 to 2.10, all 95% CIs remained above 1, and all *P* values were <0.001; I² ranged from 0% to 10.9%. The result was not materially influenced by any single study (**Figure 7**).

**Figure 7:**
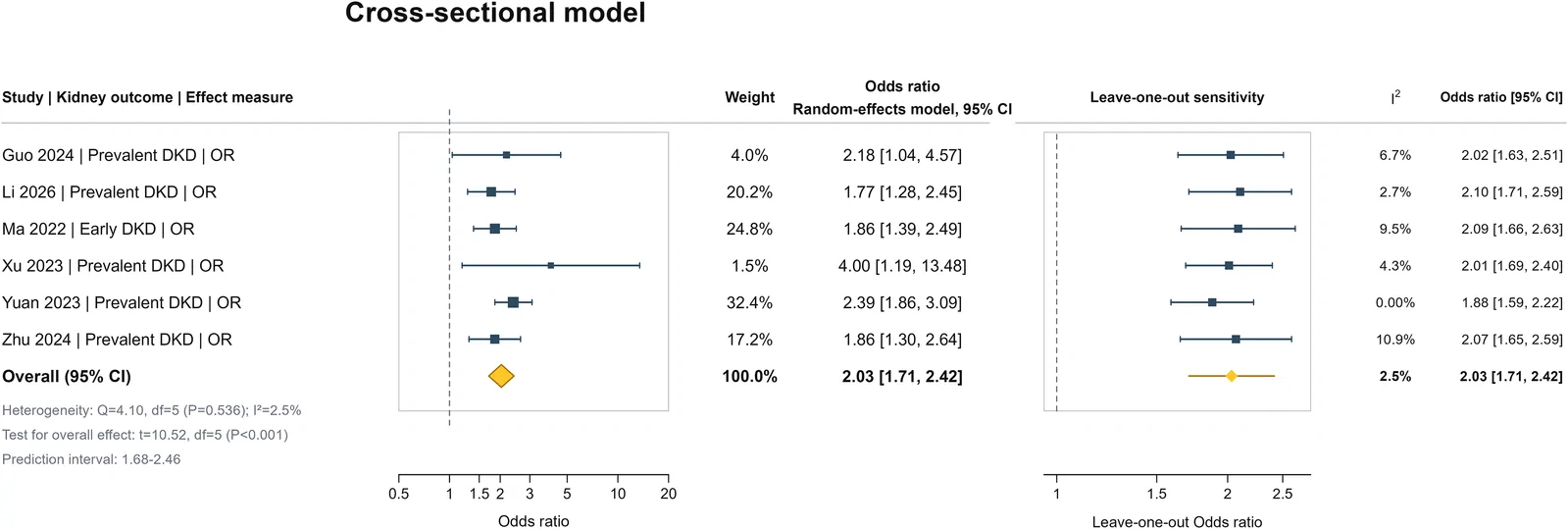
Cross-sectional association between remnant cholesterol and prevalent or early diabetic kidney disease. The left panel presents the random-effects model based on six cross-sectional studies. The right panel presents leave-one-out sensitivity analyses in which each study was omitted in turn and the model was refitted using the same analytical method. Squares and horizontal lines represent study-specific odds ratios and 95% confidence intervals; diamonds represent pooled estimates. The dashed vertical line indicates the null value of 1. CI, confidence interval; DKD, diabetic kidney disease; OR, odds ratio; RC, remnant cholesterol.

#### 3.2.6 Cardiometabolic adjustment sensitivity analyses

The three-domain restriction included 12 estimates from 11 articles that adjusted for blood pressure, glycemia, and adiposity. Higher RC remained associated with mixed kidney outcomes (association ratio 1.46, 95% CI 1.25–1.69, *P* < 0.001; I²=87.9%; **Figure 8A**). The longitudinal subset included 10 estimates from nine articles (HR 1.28, 95% CI 1.21–1.37, *P* < 0.001; I²=17.5%; **Figure 8A**), whereas the two-study cross-sectional subset was not statistically significant (OR 2.13, 95% CI 0.44–10.42, *P* = 0.104; I²=38.0%; **Figure 8A**).

**Figure 8:**
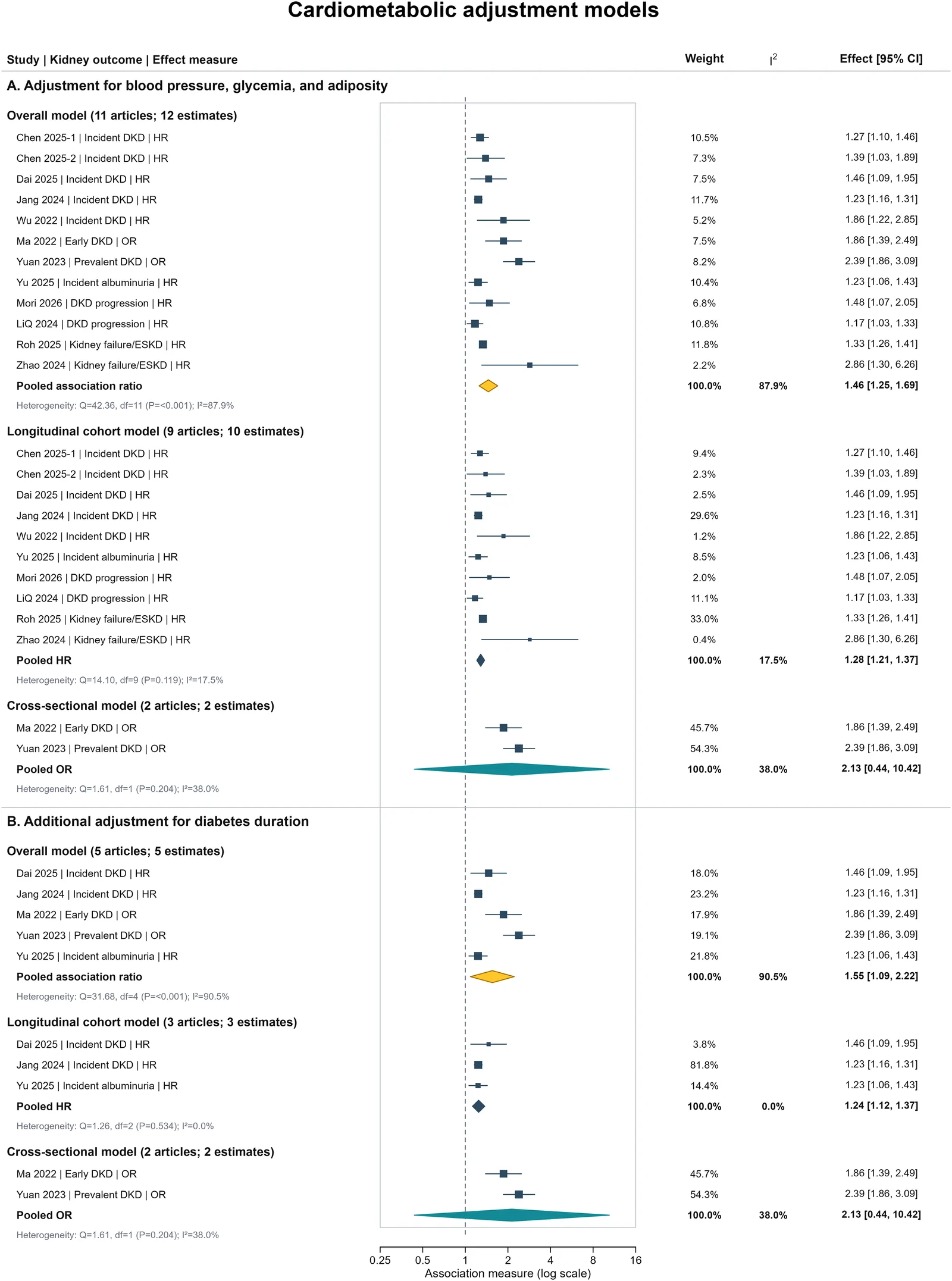
Cardiometabolic adjustment models for the association between remnant cholesterol and kidney outcomes. **(A)** Analyses restricted to estimates adjusted for blood pressure or hypertension, glycemia, and adiposity, presented for the overall study, longitudinal cohorts, and cross-sectional studies. **(B)** Analyses restricted to estimates adjusted for blood pressure or hypertension, glycemia, adiposity and diabetes duration, presented for the overall study, longitudinal cohorts, and cross-sectional studies. Squares and horizontal lines indicate study-specific estimates and 95% confidence intervals; diamonds indicate pooled estimates. CI, confidence interval; DKD, diabetic kidney disease; ESKD, end-stage kidney disease; HR, hazard ratio; OR, odds ratio; RC, remnant cholesterol.

After diabetes duration was added as a fourth required adjustment domain, five articles remained. Higher RC was associated with mixed kidney outcomes in the overall restricted model (association ratio 1.55, 95% CI 1.09–2.22, *P* = 0.027; I²=90.5%; **Figure 8B**). The longitudinal subset included three studies (HR 1.24, 95% CI 1.12–1.37, *P* = 0.011; I²≈0%; **Figure 8B**), whereas the two-study cross-sectional estimate remained nonsignificant (OR 2.13, 95% CI 0.44–10.42, *P* = 0.104; I²=38.0%; **Figure 8B**). These analyses compared different restricted study sets and did not represent within-study stepwise covariate adjustment.

#### 3.2.7 Baseline eGFR and ACR across RC categories

Four studies contributed baseline eGFR data. No significant difference was observed between the highest and lowest RC groups (MD 0.78 mL/min/1.73 m², 95% CI −2.28 to 3.84, *P* = 0.477; I²=99.7%; **Figure 9A**). Two studies contributed baseline ACR data, and the estimated ratio of medians between the highest and lowest RC groups was not statistically significant (1.15, 95% CI 0.21–6.22, *P* = 0.492; I²=94.2%; **Figure 9B**). These exploratory analyses compared baseline kidney markers and should not be interpreted as longitudinal changes.

**Figure 9:**
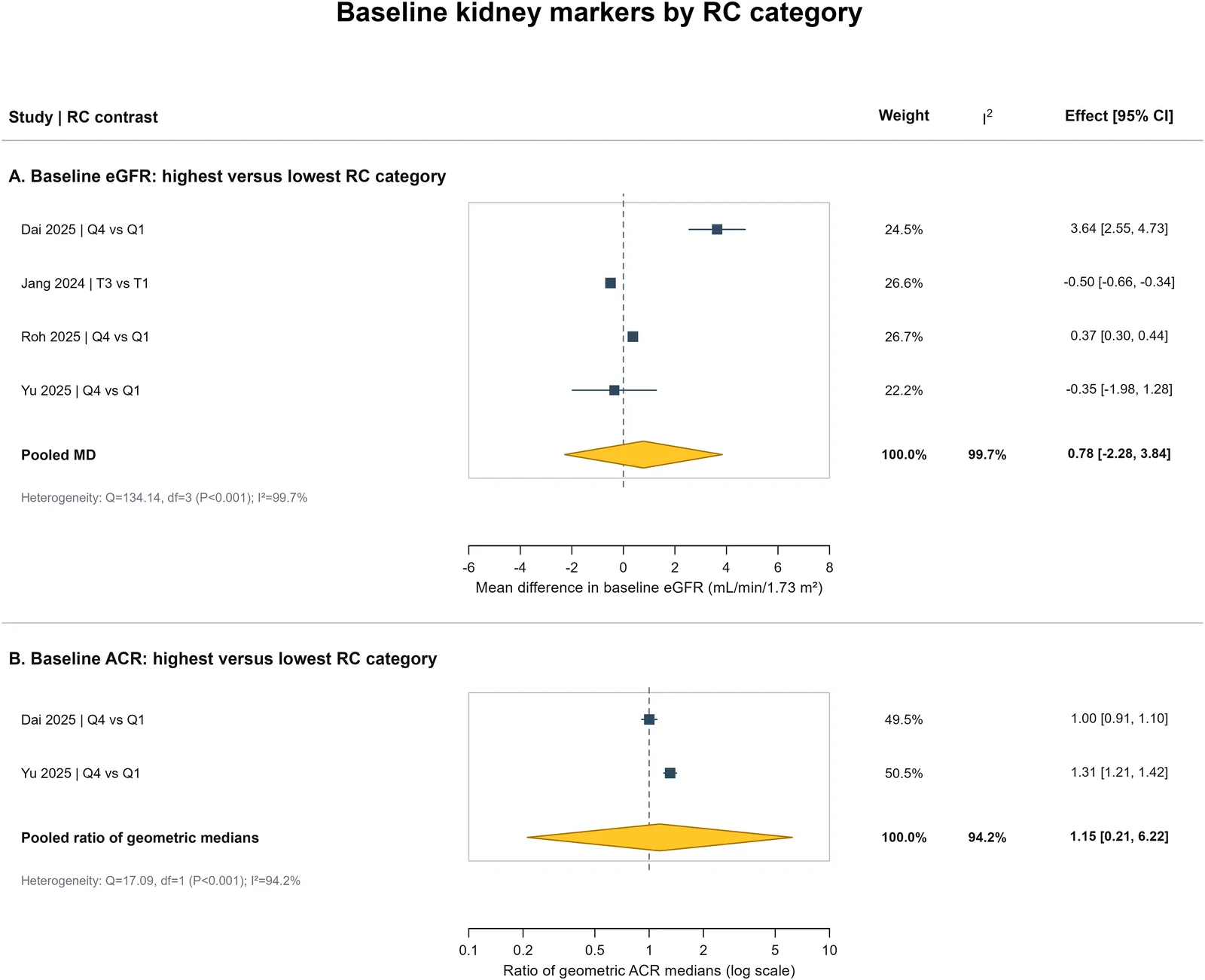
Baseline kidney markers according to remnant cholesterol category. **(A)** Mean differences in baseline estimated glomerular filtration rate between the highest and lowest RC categories. **(B)** Ratios of baseline albumin-to-creatinine ratio medians between the highest and lowest RC categories. Squares and horizontal lines indicate study-specific estimates and 95% confidence intervals; diamonds indicate pooled estimates. ACR, albumin-to-creatinine ratio; CI, confidence interval; eGFR, estimated glomerular filtration rate; MD, mean difference; RC, remnant cholesterol.

## 4 Discussion

The pathogenesis of DKD is multifactorial. In addition to hyperglycemic injury, the kidneys are exposed to chronic lipotoxic stress. Excess fatty acids may disrupt cellular integrity through oxidative stress, lipid peroxidation, and inflammatory activation, thereby contributing to renal tubular epithelial cell injury (27). RC represents the cholesterol content of TRL remnant particles and captures metabolic risk not fully reflected by traditional lipid parameters. In patients with T2DM, increased circulating free fatty acids, enhanced hepatic very-low-density lipoprotein production, and impaired lipoprotein lipase-mediated clearance jointly promote RC accumulation (28, 29). Higher RC may therefore coexist with insulin resistance, hyperglycemia, obesity, hypertension, and other metabolic abnormalities implicated in renal microvascular injury (30, 31). Our study included 17 articles, 18 independent estimates, and 2,817,820 participants. The exploratory all-study model showed an overall association between higher RC and adverse kidney outcomes. Longitudinal analyses linked higher RC to incident DKD and showed positive but nonsignificant pooled estimates for DKD progression and kidney failure/ESKD. The cross-sectional model further showed an association between higher RC and prevalent or early DKD. The overall and longitudinal associations remained evident in analyses restricted to estimates adjusted for blood pressure, glycemia, adiposity, and diabetes duration. These findings support the potential value of RC as a complementary marker for kidney-risk stratification in adults with T2DM.

Compared with triglycerides, RC more directly represents the cholesterol burden carried by triglyceride-rich, apolipoprotein B-containing remnant particles (32, 33). Evidence suggests that RC may contribute to vascular injury and inflammation. Epidemiological studies have associated higher RC with ischemic heart disease and low-grade inflammation (34, 35). *In vitro* experiments further showed that remnant lipoproteins increased the expression of inflammatory chemokines in human vascular endothelial cells through redox-sensitive pathways, suggesting potential roles in endothelial activation, leukocyte adhesion, and prothrombotic responses (36). Among diabetic microvascular complications, higher RC has also been associated with diabetic retinopathy and greater disease severity, indicating that these complications may share common pathophysiological pathways in T2DM (37).

DKD develops through multiple interacting mechanisms, including glucolipotoxicity-induced oxidative injury, accumulation of advanced glycation end products, dysregulation of the renin–angiotensin system, inflammation, and fibrosis, which collectively damage glomerular endothelial cells, podocytes, mesangial cells, and tubular epithelial cells (38, 39). Experimental studies indicate that abnormal lipid accumulation disrupts mitochondrial and endoplasmic reticulum function, increases oxidative stress, impairs autophagy, and promotes apoptosis in podocytes and proximal tubular cells (40, 41). Analyses of kidney tissue from patients with T2DM and DKD have demonstrated marked lipid deposition, accompanied by downregulation of fatty acid oxidation and cholesterol efflux pathways, upregulation of lipid-uptake receptors, and increased expression of inflammatory and fibrotic genes (42). Moreover, insulin resistance in T2DM promotes VLDL overproduction and delays remnant clearance, prolonging the residence time of RC-containing particles in the circulation and potentially increasing glomerular endothelial exposure, oxidative stress, and inflammatory activation (28, 43, 44). The association of apolipoprotein E-related remnant metabolism with DKD, together with observations that declining kidney function is accompanied by remnant accumulation, further suggests a potentially bidirectional relationship between RC and kidney injury (45, 46). RC may therefore help identify residual lipid burden not adequately captured by conventional lipid measures, providing a biologically plausible explanation for its association with the onset and progression of DKD.

The findings of this study provide partial clinical support for the experimental evidence described above: higher RC was associated with incident DKD, while the estimates for DKD progression and kidney failure/ESKD were positive but did not reach statistical significance. A single study also reported an increased risk of incident albuminuria with higher RC. This suggests that RC may contribute to the development and progression of DKD by aggravating insulin resistance, inflammation, and oxidative stress and by damaging the glomerular endothelium and podocyte barrier (35, 36, 46, 47). The direct continuous analysis included three cohorts from two articles and found no statistically significant association per 0.1 mmol/L increase in RC (HR 1.02, 95% CI 0.98–1.07; *P* = 0.197; I^2^ = 81.4%). The highest-versus-lowest analysis included five cohorts from four articles and showed a significant association (HR 1.27, 95% CI 1.15–1.41; *P* = 0.0029; I^2^ = 7.4%). The exploratory spline analysis, reconstructed from categorical data from the same five cohorts, also showed a significant overall association (*P* = 0.0128), although the test for nonlinearity was not significant (*P* = 0.340). These analyses evaluated different exposure contrasts and statistical hypotheses. The continuous model estimated an average log-linear association per 0.1 mmol/L across only three cohorts with different source populations and RC distributions, making its pooled estimate and I^2^ sensitive to between-cohort differences. The highest-versus-lowest model included two additional cohorts and compared broader study-specific RC contrasts, with more consistent estimates across studies, which may partly explain its significant result and lower heterogeneity. The significant overall spline test supported an association across the reconstructed RC range but did not quantify a single per-unit effect. The nonsignificant test for nonlinearity provided no evidence that the association departed from linearity or that a common threshold was present. Taken together, the findings suggest an overall association across the observed RC range but do not define a consistent per-unit effect, a nonlinear pattern, or a common clinical threshold. Higher RC was also associated with prevalent or early DKD in the cross-sectional analysis. Baseline-status analyses yielded positive estimates in cohorts without baseline DKD, with established DKD, and with mixed baseline status, although the estimate for kidney failure/ESKD among patients with established DKD was not statistically significant.

Available clinical evidence is broadly aligned with the findings of this study. Yang et al. reported that elevated RC was associated with a higher risk of cardiovascular disease (48). For kidney outcomes, Wu et al. evaluated both baseline and cumulative RC and found that higher RC was associated with incident DKD. In a nationwide cohort of patients with newly diagnosed T2DM, Jang et al. observed an increased risk of incident CKD among those with higher RC levels, and Chen et al. and Dai et al. subsequently reported directionally similar findings in other T2DM populations (6, 8, 14, 15). Among patients with established kidney disease, Li et al. and Mori et al. reported associations of higher RC with kidney function decline or disease progression (12, 19). In a cohort of more than 2.5 million patients with T2DM, Roh et al. observed progressively greater ESKD risk with increasing RC levels, while Zhao et al. similarly reported an association between RC and the progression of DKD to ESKD. Yu et al. further associated higher RC with adverse kidney outcomes, including kidney function decline and incident albuminuria (13, 17, 18). Previous cross-sectional studies also support an association between RC and diabetic kidney injury. Zhu et al. and Yuan et al. associated higher RC with prevalent DKD and greater CKD severity, while Eto et al. suggested that abnormalities in apolipoprotein E-related remnant metabolism may be involved in DKD. Sonoda et al. and Matsushima-Nagata et al. also observed associations between declining kidney function and higher circulating RC levels (21, 23, 45, 46, 49). In addition, a previous systematic review reported an association between higher RC and CKD, including an association with greater CKD risk in the T2DM subgroup, although substantial heterogeneity was present (9).

RC can be calculated from routine lipid measurements at low cost, supporting its potential use in clinical practice as a complementary measure of TRL-related residual risk after LDL-C targets have been achieved (50). In patients with T2DM, evaluating RC alongside TG, TC, LDL-C, eGFR, and ACR may enable a more comprehensive assessment of metabolic abnormalities and residual kidney risk. Current lipid-lowering therapies generally have a smaller effect on RC than on LDL-C. A meta-regression of trials evaluating statins, ezetimibe, and PCSK9 inhibitors showed that each 1 mmol/L reduction in LDL-C was accompanied by an average RC reduction of approximately 0.19 mmol/L, while a 20% reduction in LDL-C corresponded to an average RC reduction of approximately 11% (51). These findings suggest that conventional lipid-lowering therapy may not fully address elevated RC levels. However, evidence from intervention studies remains inconclusive. The FIELD, ACCORD, and PROMINENT trials reported inconsistent effects of modifying TRL metabolism on kidney and cardiovascular outcomes (52–55). By contrast, emerging therapies targeting apolipoprotein C-III or ANGPTL3 have reduced RC by approximately 38%–70%, providing new approaches for directly modifying remnant lipoprotein metabolism (56–58). Future clinical trials should prespecify eGFR decline and ACR progression as kidney outcomes and determine whether lowering RC provides additional kidney benefits in patients with T2DM.

Lipid-lowering and renoprotective therapies may influence RC and DKD, but adjustment for medication use varied across the included studies. Chen et al. (14) and Zhu et al. (23) adjusted for lipid-lowering therapy; Roh et al. (18) and Dai et al. (15) adjusted for statins/fibrates and antidiabetic medications, with the latter including SGLT2 inhibitors/GLP-1 receptor agonists. Li et al. (12, 25) adjusted for lipid-lowering therapy, SGLT2 inhibitors, and ACEIs/ARBs, whereas Zhao et al. (13) adjusted for statins, insulin, and renin–angiotensin–aldosterone system inhibitors. Other studies used broader treatment variables: diabetes medications in Wu et al. (8), antihypertensive therapy in Yuan et al. (21), and treatment regimens in Yu et al. (17). The remaining studies did not report adjustment for specific drug classes or provided insufficient detail (6, 16, 20, 22, 24, 26); Mori et al. (19) included medication variables only in additional sensitivity analyses. Our meta-analysis pooled the published adjusted estimates, but the absence of individual participant data and inconsistent reporting of medication classes, doses, treatment duration, and treatment changes prevented uniform additional adjustment for medication use. Drug-related residual confounding therefore remains possible. Future studies should report medication use consistently and account for treatment changes during follow-up.

The high heterogeneity in the exploratory all-study model (I^2^ = 87.6%) may partly reflect differences in study design, effect measures, kidney outcomes, RC exposure contrasts, and covariate adjustment. To examine these differences, we analyzed longitudinal and cross-sectional studies separately and further grouped longitudinal estimates by kidney outcome. Heterogeneity decreased to 42.9% in the longitudinal model and 2.5% in the cross-sectional model. Outcome-specific longitudinal analyses yielded I^2^ values of 7.4% for incident DKD, 0.1% for kidney function decline or progression, and 69.5% for kidney failure/ESKD. The higher heterogeneity in the kidney failure/ESKD analysis after inclusion of Shang et al. may reflect differences in baseline kidney severity, outcome definitions, and RC exposure contrasts. Leave-one-out analyses showed that the longitudinal association remained stable, although heterogeneity persisted after individual estimates were omitted (I^2^ = 17.1%–52.1%). Restricting the longitudinal analysis to estimates adjusted for blood pressure, glycemia, and adiposity reduced I^2^ to 17.5%, suggesting that differences in covariate adjustment may also contribute. The all-study estimate should therefore be interpreted as an exploratory summary, with greater emphasis on the design- and outcome-specific results. Future studies should standardize RC measurement and reporting scales, clearly distinguish baseline kidney status, and use consistent kidney outcome definitions and covariate adjustment to reduce methodological heterogeneity.

This study has several limitations. Although most studies used multivariable adjustment, all included evidence was observational; therefore, residual confounding, reverse causation, and selective reporting cannot be excluded, while the cross-sectional studies could not establish temporality. The evidence base was also limited for several outcomes, with only three studies for DKD progression, four for kidney failure/ESKD, one for incident albuminuria, and two for baseline ACR, reducing the precision of these estimates. The baseline-status subgroups also contained few estimates and included different RC exposure contrasts and effect measures. Generalizability may also be limited because most participants were from East Asia, with relatively few studies from the United Kingdom and the United States. Finally, incomplete reporting of lipid-lowering therapies, SGLT2 inhibitors, GLP-1 receptor agonists, and renin–angiotensin system blockers may have introduced additional confounding.

Overall, the available evidence supports an observational association between RC and the development and progression of DKD in patients with T2DM, although its causal relevance and clinical utility require confirmation in prospective studies.

## 5 Conclusion

In conclusion, we found that higher RC was associated with incident DKD and adverse longitudinal kidney outcomes overall in adults with T2DM, whereas the associations with DKD progression and kidney failure/ESKD did not reach statistical significance. These findings suggest that RC may have value as a complementary marker of adverse kidney outcomes in adults with T2DM. However, no common RC threshold was identified, and the available evidence was observational; therefore, these findings require confirmation in standardized prospective studies.

## Data Availability

The original contributions presented in the study are included in the article/**Supplementary Material**. Further inquiries can be directed to the corresponding author.

## References

[1] Lin X Xu Y Pan X Xu J Ding Y Sun X . Global, regional, and national burden and trend of diabetes in 195 countries and territories: an analysis from 1990 to 2025. Sci Rep. (2020) 10:14790. doi: 10.1038/s41598-020-71908-9 32901098 PMC7478957

[2] Federation ID . Global Diabetes Data & Statistics (2025). Available online at: https://diabetesatlas.org/ (Accessed August 1, 2026).

[3] Ruiz-Ortega M Rayego-Mateos S Lamas S Ortiz A Rodrigues-Diez R . Targeting the progression of chronic kidney disease. Nat Rev Nephrol. (2020) 16:269–88. doi: 10.1038/s41581-019-0248-y 32060481

[4] Selby N Taal M . An updated overview of diabetic nephropathy: Diagnosis, prognosis, treatment goals and latest guidelines. Diabetes Obes Metab. (2020) 22:3–15. doi: 10.1111/dom.14007 32267079

[5] Skudder-Hill L Sequeira-Bisson I Ko J Cho J Poppitt S Petrov M . Remnant cholesterol, but not low-density lipoprotein cholesterol, is associated with intra-pancreatic fat deposition. Diabetes Obes Metab. (2023) 25:3337–46. doi: 10.1111/dom.15233 37529874

[6] Jang S Kang M Song E Jang A Choi K Baik S . Remnant cholesterol is an independent risk factor for the incidence of chronic kidney disease in newly-diagnosed type 2 diabetes: A nationwide population-based study. Diabetes Res Clin Pract. (2024) 210:111639. doi: 10.1016/j.diabres.2024.111639 38548106

[7] Byrne P Lin T Jones M Demasi M Saif-Ur-Rahman K Dubroff R . The association between remnant cholesterol and cardiovascular events: A systematic review and meta-regression. J Clin Lipidol. (2026) 20:1339–51. doi: 10.1016/j.jacl.2026.03.026 42031588

[8] Wu Z Yu S Zhu Q Li Z Zhang H Kang X . Association of baseline and cumulative remnant cholesterol with incidence of diabetic nephropathy: A longitudinal cohort study. Diabetes Res Clin Pract. (2022) 191:110079. doi: 10.1016/j.diabres.2022.110079 36099974

[9] Karakasis P Patoulias D Rizzo M Fragakis N Mantzoros C . Association between remnant cholesterol and chronic kidney disease: Systematic review and meta-analysis. Diabetes Obes Metab. (2025) 27:2573–83. doi: 10.1111/dom.16258 39950216 PMC11964997

[10] Page M McKenzie J Bossuyt P Boutron I Hoffmann T Mulrow C . The PRISMA 2020 statement: an updated guideline for reporting systematic reviews. BMJ. (2021) 372:n71. doi: 10.1136/bmj.n71 33782057 PMC8005924

[11] Guyatt G Oxman A Vist G Kunz R Falck-Ytter Y Alonso-Coello P . GRADE: an emerging consensus on rating quality of evidence and strength of recommendations. BMJ. (2008) 336:924–6. doi: 10.1136/bmj.39489.470347.AD 18436948 PMC2335261

[12] Li Q Wang T Shao X Fan X Lin Y Cui Z . Association of remnant cholesterol with renal function and its progression in patients with type 2 diabetes related chronic kidney disease. Front Endocrinol (Lausanne). (2024) 15:1331603. doi: 10.3389/fendo.2024.1331603 39027471 PMC11254661

[13] Zhao Y Liu K Zou Y Wu Y Yang J Xiao X . Remnant cholesterol and the risk of diabetic nephropathy progression to end-stage kidney disease in patients with type 2 diabetes mellitus: a longitudinal cohort study. Endocrine. (2024) 86:994–1002. doi: 10.1007/s12020-024-03948-4 39126619 PMC11554765

[14] Chen Z Shen Y Chen X Hu J Mao L Lan R . High remnant cholesterol is associated with the development of diabetic kidney disease in patients with type 2 diabetes. Diabetes Metab Syndr Obes. (2025) 18:3563–73. doi: 10.2147/DMSO.S542648 40994793 PMC12456313

[15] Dai Y Pan Q Yu Y Ma Y Chen G Wang H . Remnant cholesterol for diabetic kidney disease risk stratification in type 2 diabetes: a machine learning-based prevention tool. Front Nutr. (2025) 12:1697943. doi: 10.3389/fnut.2025.1697943 41306673 PMC12643847

[16] Da J Dong R Li Q Yang Y Yuan J Luo J . The correlation of lipid profile and serum osmolality with kidney progression in patients with type 2 diabetes mellitus. Zhonghua Yi Xue Za Zhi. (2025) 105:888–93. doi: 10.3760/cma.j.cn112137-20241111-02522 40113412

[17] Yu P Yuan Q Huang L Tao L Peng Z Pu J . The prognostic value of remnant cholesterol to adverse renal outcomes in patients with type 2 diabetes. Diabetol Metab Syndr. (2025) 17:52. doi: 10.1186/s13098-025-01617-8 39940009 PMC11823253

[18] Roh E Heo J Jung H Han K-D Kang J Lee S . Impact of remnant cholesterol on the risk for end-stage renal disease in type 2 diabetes mellitus: a nationwide population-based cohort study. Diabetes Metab J. (2025) 49:1106–15. doi: 10.4093/dmj.2024.0406 40395137 PMC12436028

[19] Mori T Yamamoto Y Hanai K Yamashige Y Murata H Shinozaki T . Remnant cholesterol and kidney disease progression in type 2 diabetes: A retrospective cohort study. J Clin Lipidol. (2025) 20:135–44. doi: 10.1016/j.jacl.2025.10.075 41298175

[20] Qunwei M Ying Z Limei K Li S Changjiang Y Xiaoyan Z . Predictive effect of remnant cholesterol on early diabetic kidney disease. Chin J Diabetes Mellitus. (2022) 14:1264–71. doi: 10.3760/cma.j.cn1115791-20220326-0013030704229

[21] Yuan Y Zhou X Ji L . Association between remnant cholesterol level and severity of chronic kidney disease in patients with type 2 diabetes. J Diabetes Complications. (2023) 37:108585. doi: 10.1016/j.jdiacomp.2023.108585 37633073

[22] Hui X Shuang L Youshuo L . Association between serum remnant cholesterol and diabetic kidney disease in elderly patients with type 2 diabetes mellitus. Chin J Geriatrics. (2023) 42:399–403. doi: 10.3760/cma.j.issn.0254-9026.2023.04.00430704229

[23] Zhu W Liu Q Liu F Jiao C Zhang L Xie H . High remnant cholesterol as a risk factor for developing chronic kidney disease in patients with prediabetes and type 2 diabetes: a cross-sectional study of a US population. Acta Diabetol. (2024) 61:735–43. doi: 10.1007/s00592-024-02249-6 38436703 PMC11101368

[24] Tianyang G Hui Z Yingying C . A study on correlation between triglycerides glucose index, remnant cholesterol and diabetic nephropathy. Chin J Clin Lab Sci. (2024) 42:256–60. doi: 10.13602/j.cnki.jcls.2024.04.04

[25] Li Y Xu Z Xiao D Yun X Liu C Xiao Q . Exploring the relationship between remnant cholesterol and diabetic kidney disease in Chinese type 2 diabetes patients. Front Nutr. (2026) 13:1793007. doi: 10.3389/fnut.2026.1793007 41983069 PMC13070960

[26] Shang H Zang D Yue Q Ma J Xu L Ma L . Associations of remnant cholesterol and apolipoprotein B with kidney function decline and progression to kidney failure in hospitalized patients with type 2 diabetic kidney disease: a retrospective cohort study. BMC Nephrol. (2026). doi: 10.1186/s12882-026-05307-z38164791

[27] Noels H Lehrke M Vanholder R Jankowski J . Lipoproteins and fatty acids in chronic kidney disease: molecular and metabolic alterations. Nat Rev Nephrol. (2021) 17:528–42. doi: 10.1038/s41581-021-00423-5 33972752

[28] Hirano T . Pathophysiology of diabetic dyslipidemia. J Atheroscler Thromb. (2018) 25:771–82. doi: 10.5551/jat.RV17023 29998913 PMC6143775

[29] Vergès B . Pathophysiology of diabetic dyslipidaemia: where are we? Diabetologia. (2015) 58:886–99. doi: 10.1007/s00125-015-3525-8 25725623 PMC4392164

[30] Li B Liu Y Zhou X Chen L Yan L Tang X . Remnant cholesterol is more positively related to diabetes, prediabetes, and insulin resistance than conventional lipid parameters and lipid ratios: A multicenter, large sample survey. J Diabetes. (2024) 16:e13592. doi: 10.1111/1753-0407.13592 39136535 PMC11320755

[31] Huh J Roh E Lee S Ihm S-H Han K-D Kang J . Remnant cholesterol is an independent predictor of type 2 diabetes: a nationwide population-based cohort study. Diabetes Care. (2023) 46:305–12. doi: 10.2337/dc22-1550 36469354

[32] Cohn J Marcoux C Davignon J . Detection, quantification, and characterization of potentially atherogenic triglyceride-rich remnant lipoproteins. Arterioscler Thromb Vasc Biol. (1999) 19:2474–86. doi: 10.1161/01.atv.19.10.2474 10521378

[33] Ginsberg H Packard C Chapman M Borén J Aguilar-Salinas C Averna M . Triglyceride-rich lipoproteins and their remnants: metabolic insights, role in atherosclerotic cardiovascular disease, and emerging therapeutic strategies-a consensus statement from the European Atherosclerosis Society. Eur Heart J. (2021) 42:4791–806. doi: 10.1093/eurheartj/ehab551 34472586 PMC8670783

[34] Varbo A Benn M Tybjærg-Hansen A Jørgensen A Frikke-Schmidt R Nordestgaard B . Remnant cholesterol as a causal risk factor for ischemic heart disease. J Am Coll Cardiol. (2012) 61:427–36. doi: 10.1016/j.jacc.2012.08.1026 23265341

[35] Varbo A Benn M Tybjærg-Hansen A Nordestgaard B . Elevated remnant cholesterol causes both low-grade inflammation and ischemic heart disease, whereas elevated low-density lipoprotein cholesterol causes ischemic heart disease without inflammation. Circulation. (2013) 128:1298–309. doi: 10.1161/CIRCULATIONAHA.113.003008 23926208

[36] Doi H Kugiyama K Oka H Sugiyama S Ogata N Koide S . Remnant lipoproteins induce proatherothrombogenic molecules in endothelial cells through a redox-sensitive mechanism. Circulation. (2000) 102:670–6. doi: 10.1161/01.cir.102.6.670 10931808

[37] Shan Y Wang Q Zhang Y Tong X Pu S Xu Y . High remnant cholesterol level is relevant to diabetic retinopathy in type 2 diabetes mellitus. Lipids Health Dis. (2022) 21:12. doi: 10.1186/s12944-021-01621-7 35057797 PMC8772129

[38] Forbes J Cooper M . Mechanisms of diabetic complications. Physiol Rev. (2013) 93:137–88. doi: 10.1152/physrev.00045.2011 23303908

[39] Alicic R Rooney M Tuttle K . Diabetic kidney disease: challenges, progress, and possibilities. Clin J Am Soc Nephrol. (2017) 12:2032–45. doi: 10.2215/CJN.11491116 28522654 PMC5718284

[40] Schelling J . The contribution of lipotoxicity to diabetic kidney disease. Cells. (2022) 11:3236. doi: 10.3390/cells11203236 36291104 PMC9601125

[41] Wang Y Liu T Wu Y Wang L Ding S Hou B . Lipid homeostasis in diabetic kidney disease. Int J Biol Sci. (2024) 20:3710–24. doi: 10.7150/ijbs.95216 39113692 PMC11302873

[42] Herman-Edelstein M Scherzer P Tobar A Levi M Gafter U . Altered renal lipid metabolism and renal lipid accumulation in human diabetic nephropathy. J Lipid Res. (2013) 55:561–72. doi: 10.1194/jlr.P040501 24371263 PMC3934740

[43] Ohnishi H Saitoh S Takagi S Ohata J Isobe T Kikuchi Y . Relationship between insulin-resistance and remnant-like particle cholesterol. Atherosclerosis. (2002) 164:167–70. doi: 10.1016/s0021-9150(02)00057-6 12119206

[44] Liu L Wen T Zheng X Yang D-G Zhao S-P Xu D-Y . Remnant-like particles accelerate endothelial progenitor cells senescence and induce cellular dysfunction via an oxidative mechanism. Atherosclerosis. (2008) 202:405–14. doi: 10.1016/j.atherosclerosis.2008.05.024 18582890

[45] Eto M Saito M Okada M Kume Y Kawasaki F Matsuda M . Apolipoprotein E genetic polymorphism, remnant lipoproteins, and nephropathy in type 2 diabetic patients. Am J Kidney Dis. (2002) 40:243–51. doi: 10.1053/ajkd.2002.34502 12148096

[46] Sonoda M Shoji T Kimoto E Okute Y Shima H Naganuma T . Kidney function, cholesterol absorption and remnant lipoprotein accumulation in patients with diabetes mellitus. J Atheroscler Thromb. (2013) 21:346–54. 10.5551/jat.20594 24335047

[47] Yoshioka K Hirakawa Y Kurano M Ube Y Ono Y Kojima K . Lysophosphatidylcholine mediates fast decline in kidney function in diabetic kidney disease. Kidney Int. (2021) 101:510–26. doi: 10.1016/j.kint.2021.10.039 34856312

[48] Yang X Zhang B Cheng Y Fu S Jin H . Association of remnant cholesterol with risk of cardiovascular disease events, stroke, and mortality: a systemic review and meta-analysis. Atherosclerosis. (2023) 371:21–31. doi: 10.1016/j.atherosclerosis.2023.03.012 36966562

[49] Matsushima-Nagata K Matsumura T Kondo Y Anraku K Fukuda K Yamanaka M . Significance of circulating remnant lipoprotein cholesterol levels measured by homogeneous assay in patients with type 2 diabetes. Biomolecules. (2023) 13:468. doi: 10.3390/biom13030468 36979403 PMC10099722

[50] Langlois M Nordestgaard B Langsted A Chapman M Aakre K Baum H . Quantifying atherogenic lipoproteins for lipid-lowering strategies: consensus-based recommendations from EAS and EFLM. Clin Chem Lab Med. (2020) 58:496–517. doi: 10.1515/cclm-2019-1253 31855562

[51] Nordestgaard AT Nordestgaard BG . Remnant cholesterol lowering in cardiovascular disease risk reduction in statin, ezetimibe, and PCSK9 inhibitor trials: meta-regression analyses. Eur J Prev Cardiol. (2025) zwaf337. doi: 10.1093/eurjpc/zwaf337 40504878

[52] Davis TME Ting R Best JD Donoghoe MW Drury PL Sullivan DR . Effects of fenofibrate on renal function in patients with type 2 diabetes mellitus: the Fenofibrate Intervention and Event Lowering in Diabetes (FIELD) Study. Diabetologia. (2010) 54:280–90. doi: 10.1007/s00125-010-1951-1 21052978

[53] Mottl AK Buse JB Ismail-Beigi F Sigal RJ Pedley CF Papademetriou V . Long-term effects of intensive glycemic and blood pressure control and fenofibrate use on kidney outcomes. Clin J Am Soc Nephrol. (2018) 13:1693–702. doi: 10.2215/CJN.06200518 30361335 PMC6237052

[54] Das Pradhan A Glynn RJ Fruchart J-C MacFadyen JG Zaharris ES Everett BM . Triglyceride lowering with pemafibrate to reduce cardiovascular risk. N Engl J Med. (2022) 387:1923–34. doi: 10.1056/NEJMoa2210645 36342113

[55] Araki E Yamashita S Arai H Yokote K Satoh J Inoguchi T . Effects of pemafibrate, a novel selective PPARα modulator, on lipid and glucose metabolism in patients with type 2 diabetes and hypertriglyceridemia: a randomized, double-blind, placebo-controlled, phase 3 trial. Diabetes Care. (2018) 41:538–46. doi: 10.2337/dc17-1589 29298800

[56] Gaudet D Karwatowska-Prokopczuk E Baum SJ Hurh E Kingsbury J Bartlett VJ . Vupanorsen, an N-acetyl galactosamine-conjugated antisense drug to ANGPTL3 mRNA, lowers triglycerides and atherogenic lipoproteins in patients with diabetes, hepatic steatosis, and hypertriglyceridaemia. Eur Heart J. (2020) 41:3936–45. doi: 10.1093/eurheartj/ehaa689 32860031 PMC7750927

[57] Zimerman A Wiviott SD Park J-G Murphy SA Ran X Bramson CR . Reductions in remnant cholesterol and VLDL cholesterol through inhibition of ANGPTL3 protein synthesis: an analysis from the TRANSLATE-TIMI 70 trial. Eur J Prev Cardiol. (2024) 31:1216–23. doi: 10.1093/eurjpc/zwae090 38484368

[58] Ballantyne CM Vasas S Azizad M Clifton P Rosenson RS Chang T . Plozasiran, an RNA interference agent targeting APOC3, for mixed hyperlipidemia. N Engl J Med. (2024) 391:899–912. doi: 10.1056/NEJMoa2404143 38804517

