# Supplementary material for: Association of remnant cholesterol with kidney outcomes in adults with type 2 diabetes: a systematic review and meta-analysis
Source: Front Endocrinol (Lausanne). 2026 Oct 16;17:1969361. doi: 10.3389/fendo.2026.1969361 (PMC13719300; doi:10.3389/fendo.2026.1969361)
Supplement: Supplementary file 1 [file DataSheet1.docx]

Supplementary material

**Contents**

[**Table S1.** Newcastle-Ottawa Scale forms, study-level assessment	1](#_Toc241184824)

[A. Newcastle-Ottawa Scale source forms	1](#_Toc241184825)

[B. Study-level Newcastle-Ottawa Scale assessment	4](#_Toc241184826)

[**Figure S1.** Graphical summary of Newcastle-Ottawa Scale quality assessment of included studies.	5](#_Toc241184827)

[**Table S2.** Study-level covariate adjustment and sensitivity analyses	6](#_Toc241184828)

[**Table S3.** GRADE summary of findings	7](#_Toc241184829)

[**Table S4.** Continuous and dose-response analyses for incident DKD	8](#_Toc241184830)

# **Table S1.** Newcastle-Ottawa Scale forms, study-level assessment

## A. Newcastle-Ottawa Scale source forms

| **D. The Newcastle-Ottawa Scale (NOS) for cohort study**  **Website:** http://www.ohri.ca/programs/clinical_epidemiology/oxford.asp | | |
| --- | --- | --- |
| Major Components | Response options | |
| **Selection** | | |
| 1. Representativeness of the exposed cohort  1) truly representative of the average _______________ (describe) in the community  2) somewhat representative of the average ______________ in the community  3) selected group of users eg nurses, volunteers  4) no description of the derivation of the cohort | ☆  ☆  /  / | |
| 2. Selection of the non exposed cohort  1) drawn from the same community as the exposed cohort  2) drawn from a different source  3) no description of the derivation of the non exposed cohort | ☆  /  / | |
| 3. Ascertainment of exposure  1) secure record (eg surgical records)  2) structured interview   3) written self report  4) no description | ☆  ☆  /  / | |
| 4. Demonstration that outcome of interest was not present at start of study  1) yes  2) no | ☆  / | |
| **Comparability*** | | |
| 5. Comparability of cohorts on the basis of the design or analysis  1) study controls for _____________ (select the most important factor)  2) study controls for any additional factor (This criteria could be modified to indicate specific control for a second important factor.) | ☆  ☆ | |
| **Outcome** | | |
| 6. Assessment of outcome  1) independent blind assessment  2) record linkage  3) self report  4) no description | ☆  ☆  /  / | |
| 7. Was follow-up long enough for outcomes to occur  1) yes (select an adequate follow up period for outcome of interest)  2) no | ☆  / | |
| 8. Adequacy of follow up of cohorts  1) complete follow up - all subjects accounted for ¯  2) subjects lost to follow up unlikely to introduce bias - small number lost - > ____ % (select an adequate %) follow up, or description provided of those lost)  3) follow up rate < ____% (select an adequate %) and no description of those lost  4) no statement | ☆  ☆  /  / | |
| *, A study can be awarded a maximum of one star for each numbered item within the Selection and Exposure categories; a maximum of two stars can be given for Comparability. | | |
| **J. The Newcastle-Ottawa Scale (NOS) for case-control study**  **Website:** http://www.ohri.ca/programs/clinical_epidemiology/oxford.asp | | |
| Major Components | | Response options |
| Selection | | |
| 1. Is the case definition adequate?  1) yes, with independent validation  2) yes, eg record linkage or based on self reports  3) no description | | ☆  /  / |
| 2. Representativeness of the cases  1) consecutive or obviously representative series of cases  2) potential for selection biases or not stated | | ☆  / |
| 3. Selection of Controls  1) community controls  2) hospital controls  3) no description | | ☆  /  / |
| 4. Definition of Controls  1) no history of disease (endpoint)  2) no description of source | | ☆  / |
| Comparability* | | |
| 5. Comparability of cases and controls on the basis of the design or analysis  1) study controls for _______________ (Select the most important factor.)  2) study controls for any additional factor (This criteria could be modified to indicate specific control for a second important factor.) | | ☆  ☆ |
| Exposure | | |
| 6. Ascertainment of exposure  1) secure record (eg surgical records)  2) structured interview where blind to case/control status  3) interview not blinded to case/control status  4) written self report or medical record only  5) no description | | ☆  ☆  /  /  / |
| 7. Same method of ascertainment for cases and controls  1) yes  2) no | | ☆  / |
| 8. Non-Response rate  1) same rate for both groups  2) non respondents described  3) rate different and no designation | | ☆  /  / |
| *, A study can be awarded a maximum of one star for each numbered item within the Selection and Exposure categories; a maximum of two stars can be given for Comparability. | | |

## B. Study-level Newcastle-Ottawa Scale assessment

| **Study** | **Study design** | **NOS application** | **Selection** | **Comparability** | **Outcome/exposure** | **Total** | **Quality** |
| --- | --- | --- | --- | --- | --- | --- | --- |
| **Dai et al., 2025** | Retrospective cohort | Standard cohort NOS | 3/4 | 2/2 | 3/3 | **8/9** | High |
| **Mori et al., 2026** | Retrospective cohort | Standard cohort NOS | 3/4 | 2/2 | 3/3 | **8/9** | High |
| **Shang et al., 2026** | Retrospective cohort | Standard cohort NOS | 3/4 | 2/2 | 3/3 | **8/9** | High |
| **Chen et al., 2025** | Dual prospective cohorts | Standard cohort NOS | 4/4 | 2/2 | 2/3 | **8/9** | High |
| **Roh et al., 2025** | Nationwide retrospective cohort | Standard cohort NOS | 4/4 | 2/2 | 3/3 | **9/9** | High |
| **Da et al., 2025** | Longitudinal hospital cohort | Standard cohort NOS | 3/4 | 1/2 | 3/3 | **7/9** | High |
| **Yu et al., 2025** | Post hoc longitudinal trial cohort | Standard cohort NOS | 3/4 | 2/2 | 3/3 | **8/9** | High |
| **Zhao et al., 2024** | Longitudinal biopsy cohort | Standard cohort NOS | 3/4 | 1/2 | 3/3 | **7/9** | High |
| **Li et al., 2024** | Longitudinal cohort | Standard cohort NOS | 3/4 | 2/2 | 2/3 | **7/9** | High |
| **Jang et al., 2024** | Nationwide retrospective cohort | Standard cohort NOS | 4/4 | 2/2 | 3/3 | **9/9** | High |
| **Wu et al., 2022** | Longitudinal health-management cohort | Standard cohort NOS | 3/4 | 1/2 | 3/3 | **7/9** | High |
| **Li et al., 2026** | Multicenter cross-sectional | Adapted case-control NOS | 3/4 | 2/2 | 2/3 | **7/9** | High |
| **Zhu et al., 2024** | NHANES cross-sectional | Adapted case-control NOS | 4/4 | 2/2 | 3/3 | **9/9** | High |
| **Yuan et al., 2023** | Hospital cross-sectional | Adapted case-control NOS | 2/4 | 2/2 | 2/3 | **6/9** | Moderate |
| **Xu et al., 2023** | Elderly inpatient cross-sectional | Adapted case-control NOS | 2/4 | 1/2 | 2/3 | **5/9** | Moderate |
| **Ma et al., 2022** | Hospital cross-sectional prediction study | Adapted case-control NOS | 2/4 | 2/2 | 2/3 | **6/9** | Moderate |
| **Guo et al., 2024** | Hospital cross-sectional | Adapted case-control NOS | 2/4 | 1/2 | 2/3 | **5/9** | Moderate |

Notes: The standard cohort NOS (Section D) was applied to longitudinal studies. Because the supplied NOS did not include a cross-sectional form, the case-control NOS (Section J) was adapted for analytical cross-sectional studies by treating participants with prevalent DKD as cases and those without DKD as controls. Chen et al. reported two independent cohorts (UK Biobank, 9/9; Chinese Diabetes Registry, 8/9); the conservative article-level score of 8/9 is shown. Review-defined categories were high quality, 7-9; moderate quality, 4-6; and low quality, 0-3. Abbreviations: DKD, diabetic kidney disease; NOS, Newcastle-Ottawa Scale.

# **Figure S1.** Graphical summary of Newcastle-Ottawa Scale quality assessment of included studies.

(a) Cohort studies assessed using the standard cohort NOS. (b) Analytical cross-sectional studies assessed using the prespecified adaptation of the case-control NOS. An asterisk indicates that a star was awarded; a dash indicates that it was not awarded.

# **Table S2.** Study-level covariate adjustment and sensitivity analyses

**Panel A. Adjustment status of the selected estimate from each study/cohort**

| **Study/cohort** | **Design** | **Kidney outcome** | **Measure** | **Adiposity** | **BP/HTN** | **Glycemia** | **DM duration** | **All four** | **Covariates in the selected model** |
| --- | --- | --- | --- | --- | --- | --- | --- | --- | --- |
| Chen2025-UKB | Longitudinal cohort | Incident DKD | HR | Yes | Yes | Yes | No | No | Age, sex, eGFR, BMI, DBP, FPG, LDL-C, lipid-lowering therapy |
| Chen2025-CDR | Longitudinal cohort | Incident DKD | HR | Yes | Yes | Yes | No | No | Age, sex, eGFR, BMI, DBP, FPG, LDL-C, lipid-lowering therapy |
| Dai2025 | Longitudinal cohort | Incident DKD | HR | Yes | Yes | Yes | Yes | Yes | Age, sex, SBP, BMI, ACR, creatinine, HbA1c, diabetes duration, hypertension, lipid therapy, SGLT2i/GLP-1RA |
| Jang2024 | Longitudinal cohort | Incident DKD | HR | Yes | Yes | Yes | Yes | Yes | Age, sex, BMI, alcohol, smoking, exercise, hypertension, dyslipidemia, CCI, glucose, income, eGFR |
| Wu2022 | Longitudinal cohort | Incident DKD | HR | Yes | Yes | Yes | No | No | Age, sex, obesity, smoking, alcohol, activity, hypertension, dyslipidemia, glucose, HbA1c, diabetes medication |
| Li2026 | Analytical cross-sectional | Prevalent DKD | OR | No | Yes | No | No | No | Age, sex, smoking, diabetic complications, macrovascular comorbidities, ACEI/ARB, SGLT2i, lipid-lowering therapy |
| Ma2022 | Analytical cross-sectional | Prevalent DKD | OR | Yes | Yes | Yes | Yes | Yes | Diabetic retinopathy, diabetes duration, SBP, BMI, HbA1c, serum creatinine, and uric acid |
| Xu2023 | Analytical cross-sectional | Prevalent DKD | OR | No | Yes | No | Yes | No | Age, diabetes duration, SBP, fasting C-peptide, albumin, TG, HDL-C, uric acid, serum creatinine, UACR, and eGFR |
| Yuan2023 | Analytical cross-sectional | Prevalent DKD | OR | Yes | Yes | Yes | Yes | Yes | Age, sex, smoking, diabetes duration, BMI, SBP, HbA1c, antihypertensive treatment, and cardiovascular disease |
| Zhu2024 | Analytical cross-sectional | Prevalent DKD | OR | No | Yes | Yes | No | No | Age, sex, race, education, poverty-to-income ratio, smoking, alcohol consumption, hypertension, fasting glucose, HbA1c, albumin, neutrophil count, lymphocyte count, platelet count, NLR, and lipid-lowering medication use |
| Yu2025 | Longitudinal cohort | Incident albuminuria | HR | Yes | Yes | Yes | Yes | Yes | Age, sex, race, education, BMI, smoking, alcohol, BP, HbA1c, glucose, diabetes duration, creatinine, urinary albumin, CVD and treatment regimens |
| Da2025 | Longitudinal cohort | Kidney progression | HR | No | No | Yes | No | No | Sex, eGFR, hemoglobin, platelet count, cystatin C, uric acid, and NLR |
| Mori2026 | Longitudinal cohort | Kidney progression | HR | Yes | Yes | Yes | No | No | Age, sex, history of ischemic heart disease or stroke, BMI, SBP, HbA1c, eGFR, log-transformed UACR, and mean HDL-C and LDL-C |
| LiQ2024 | Longitudinal cohort | Kidney progression | HR | Yes | Yes | Yes | No | No | Age, sex, baseline eGFR, 24-hour urinary protein, BMI, SBP, HbA1c, hemoglobin, ACEI/ARB, SGLT2 inhibitor, GLP-1 receptor agonist, and lipid-lowering therapy |
| Roh2025 | Longitudinal cohort | Kidney failure/ESKD | HR | Yes | Yes | Yes | No | No | Age, sex, BMI, smoking, alcohol, exercise, income, hypertension, eGFR, statin/fibrate, FPG, diabetes medication, TC, TG |
| Zhao2024 | Longitudinal cohort | Kidney failure/ESKD | HR | Yes | Yes | Yes | No | No | Age, sex, BMI, smoking, hypertension, HbA1c, dyslipidemia, RAAS inhibitor use, insulin use, statin use, eGFR, proteinuria, and renal pathological features |
| Shang2026 | Longitudinal cohort | Kidney failure/ESKD | HR | No | Yes | Yes | No | No | Age, sex, baseline eGFR, urine protein grade, HbA1c, hypertension, hemoglobin, NLR, and uric acid |
| Guo2024 | Analytical cross-sectional | Prevalent DKD | OR | No | Yes | No | Yes | No | Diabetes duration, SBP, DBP, urea, creatinine, uric acid, triglycerides, TyG index, and RC |

Panel A notes: Yes indicates adjustment for the named domain. Diabetes duration was also classified as controlled for cohorts restricted to newly diagnosed T2DM. Mori 2026 was classified as not controlled because diabetes duration appeared only in a sensitivity model. BP, blood pressure; DM, diabetes mellitus; HTN, hypertension.

**Panel B. Pooled sensitivity analyses according to adjustment status**

| **Evidence set** | **Adjustment domain** | **Status** | **Articles** | **Estimates** | **Measure** | **Effect [95% CI]** | **P value** | **I2, %** | **Tau2** | **P difference** | **Interpretation** |
| --- | --- | --- | --- | --- | --- | --- | --- | --- | --- | --- | --- |
| **All studies** | **Reference model** | **All selected estimates** | **17** | **18** | **Association ratio** | **1.50 [1.32, 1.72]** | **<0.001** | **87.6** | **0.0428** | **NA** | **Mixed HR/OR and kidney outcomes** |
| All studies | Adiposity (BMI/weight/obesity) | Not controlled | 6 | 6 | Association ratio | 1.73 [1.19, 2.53] | 0.013 | 71.9 | 0.0835 | NA | Mixed HR/OR and kidney outcomes |
| All studies | Adiposity (BMI/weight/obesity) | Controlled | 11 | 12 | Association ratio | 1.46 [1.25, 1.69] | <0.001 | 87.9 | 0.0362 | 0.427 | Mixed HR/OR and kidney outcomes |
| All studies | Blood pressure/hypertension | Not controlled | 1 | 1 | Association ratio | 1.12 [1.02, 1.23] | NA | NA | NA | NA | Single estimate; no pooling |
| All studies | Blood pressure/hypertension | Controlled | 16 | 17 | Association ratio | 1.54 [1.35, 1.76] | <0.001 | 85.2 | 0.0399 | NA | Mixed HR/OR and kidney outcomes |
| All studies | Glycemia | Not controlled | 3 | 3 | Association ratio | 1.91 [1.06, 3.46] | 0.042 | 0.0 | 0.0000 | NA | Mixed HR/OR and kidney outcomes |
| All studies | Glycemia | Controlled | 14 | 15 | Association ratio | 1.46 [1.27, 1.68] | <0.001 | 88.9 | 0.0408 | 0.161 | Mixed HR/OR and kidney outcomes |
| All studies | Diabetes duration | Not controlled | 10 | 11 | Association ratio | 1.41 [1.21, 1.65] | <0.001 | 78.6 | 0.0247 | NA | Mixed HR/OR and kidney outcomes |
| All studies | Diabetes duration | Controlled | 7 | 7 | Association ratio | 1.64 [1.21, 2.23] | 0.007 | 87.1 | 0.0735 | 0.462 | Mixed HR/OR and kidney outcomes |
| All studies | All four domains | Not controlled | 12 | 13 | Association ratio | 1.46 [1.25, 1.71] | <0.001 | 79.3 | 0.0308 | NA | Mixed HR/OR and kidney outcomes |
| All studies | All four domains | Controlled | 5 | 5 | Association ratio | 1.55 [1.09, 2.22] | 0.027 | 90.5 | 0.0710 | 0.836 | Mixed HR/OR and kidney outcomes |
| **Longitudinal cohort** | **Reference model** | **All selected estimates** | **11** | **12** | **HR** | **1.27 [1.17, 1.37]** | **<0.001** | **42.9** | **0.0032** | **NA** | **Longitudinal HR overview** |
| Longitudinal cohort | Adiposity (BMI/weight/obesity) | Not controlled | 2 | 2 | HR | 1.49 [0.02, 134.06] | 0.461 | 78.3 | 0.2052 | NA | Longitudinal HR overview |
| Longitudinal cohort | Adiposity (BMI/weight/obesity) | Controlled | 9 | 10 | HR | 1.28 [1.21, 1.37] | <0.001 | 17.5 | 0.0009 | 0.173 | Longitudinal HR overview |
| Longitudinal cohort | Blood pressure/hypertension | Not controlled | 1 | 1 | HR | 1.12 [1.02, 1.23] | NA | NA | NA | NA | Single estimate; no pooling |
| Longitudinal cohort | Blood pressure/hypertension | Controlled | 10 | 11 | HR | 1.29 [1.21, 1.38] | <0.001 | 17.1 | 0.0010 | NA | Longitudinal HR overview |
| Longitudinal cohort | Glycemia | Not controlled | 0 | 0 | NA | No eligible estimate | NA | NA | NA | NA | No estimate available |
| Longitudinal cohort | Glycemia | Controlled | 11 | 12 | HR | 1.27 [1.17, 1.37] | <0.001 | 42.9 | 0.0032 | NA | Longitudinal HR overview |
| Longitudinal cohort | Diabetes duration | Not controlled | 8 | 9 | HR | 1.30 [1.13, 1.50] | 0.002 | 61.2 | 0.0091 | NA | Longitudinal HR overview |
| Longitudinal cohort | Diabetes duration | Controlled | 3 | 3 | HR | 1.24 [1.12, 1.37] | 0.011 | 0.0 | 0.0000 | 0.822 | Longitudinal HR overview |
| Longitudinal cohort | All four domains | Not controlled | 8 | 9 | HR | 1.30 [1.13, 1.50] | 0.002 | 61.2 | 0.0091 | NA | Longitudinal HR overview |
| Longitudinal cohort | All four domains | Controlled | 3 | 3 | HR | 1.24 [1.12, 1.37] | 0.011 | 0.0 | 0.0000 | 0.822 | Longitudinal HR overview |
| **Cross-sectional** | **Reference model** | **All selected estimates** | **6** | **6** | **OR** | **2.03 [1.71, 2.42]** | **<0.001** | **2.5** | **0.0009** | **NA** | **Cross-sectional prevalence OR** |
| cross-sectional | Adiposity (BMI/weight/obesity) | Not controlled | 4 | 4 | OR | 1.89 [1.43, 2.50] | 0.005 | 0.0 | 0.0000 | NA | Cross-sectional prevalence OR |
| cross-sectional | Adiposity (BMI/weight/obesity) | Controlled | 2 | 2 | OR | 2.13 [0.44, 10.42] | 0.104 | 38.0 | 0.0119 | 0.411 | Cross-sectional prevalence OR |
| cross-sectional | Blood pressure/hypertension | Not controlled | 0 | 0 | NA | No eligible estimate | NA | NA | NA | NA | No estimate available |
| cross-sectional | Blood pressure/hypertension | Controlled | 6 | 6 | OR | 2.03 [1.71, 2.42] | <0.001 | 2.5 | 0.0009 | NA | Cross-sectional prevalence OR |
| cross-sectional | Glycemia | Not controlled | 3 | 3 | OR | 1.91 [1.06, 3.46] | 0.042 | 0.0 | 0.0000 | NA | Cross-sectional prevalence OR |
| cross-sectional | Glycemia | Controlled | 3 | 3 | OR | 2.07 [1.42, 3.02] | 0.014 | 14.6 | 0.0039 | 0.688 | Cross-sectional prevalence OR |
| cross-sectional | Diabetes duration | Not controlled | 2 | 2 | OR | 1.81 [1.34, 2.45] | 0.026 | 0.0 | 0.0000 | NA | Cross-sectional prevalence OR |
| cross-sectional | Diabetes duration | Controlled | 4 | 4 | OR | 2.18 [1.63, 2.91] | 0.003 | 10.1 | 0.0052 | 0.211 | Cross-sectional prevalence OR |
| cross-sectional | All four domains | Not controlled | 4 | 4 | OR | 1.89 [1.43, 2.50] | 0.005 | 0.0 | 0.0000 | NA | Cross-sectional prevalence OR |
| cross-sectional | All four domains | Controlled | 2 | 2 | OR | 2.13 [0.44, 10.42] | 0.104 | 38.0 | 0.0119 | 0.411 | Cross-sectional prevalence OR |

Panel B notes: The all-study rows combine HRs and ORs across different kidney outcomes and are exploratory. Abbreviations: BMI, body mass index; CI, confidence interval; HR, hazard ratio; I2, proportion of total variation due to heterogeneity; NA, not applicable or not estimable; OR, odds ratio; Tau2, between-study variance; T2DM, type 2 diabetes mellitus.

# **Table S3.** GRADE summary of findings

| **Outcome** | **Design** | **Studies/ estimates** | **Participants/ note** | **Pooled effect** | **Risk of bias** | **Inconsistency** | **Indirectness** | **Imprecision** | **Publication bias** | **Other considerations** | **Certainty** | **Interpretation** |
| --- | --- | --- | --- | --- | --- | --- | --- | --- | --- | --- | --- | --- |
| Incident DKD  highest versus lowest RC | Observational longitudinal | 4 articles; 5 cohort estimates | Multiple T2DM cohorts | HR 1.27 (95% CI 1.15 to 1.41); PI 1.12 to 1.44 | Not serious | Not serious | Not serious | Not serious | Not formally assessed (<10 estimates) | Consistent association; residual confounding prevents upgrading | Low | Higher RC is probably associated with greater incident DKD risk, but causality is not established. |
| Kidney function decline or progression | Observational longitudinal | 3 studies; 3 categorical estimates | Mixed baseline kidney status and progression definitions | HR 1.17 (95% CI 1.00 to 1.37); PI 1.00 to 1.37 | Serious | Not serious | Serious | Serious | Not formally assessed (<10 estimates) | Same-direction estimates but clinical endpoint definitions and category thresholds differ | Very low | Higher RC may be associated with kidney progression, but the Hartung-Knapp interval includes the null after harmonizing exposure scale. |
| Kidney failure or ESKD | Observational longitudinal | 4 studies; 4 categorical estimates | ESKD, renal-failure composite, and first recorded eGFR <15 mL/min/1.73 m² | HR 1.56 (95% CI 0.82 to 2.97); PI 0.50 to 4.90 | Serious | Serious | Serious | Serious | Not formally assessed (<10 estimates) | Baseline kidney severity, kidney-failure definitions, and RC contrasts differed across studies | Very low | The pooled estimate was positive but imprecise and not statistically significant; certainty remains very low. |
| Incident albuminuria | Observational longitudinal | 1 study; 1 estimate | ACCORD secondary cohort | HR 1.23 (95% CI 1.06 to 1.43) | Serious | Not applicable | Not serious | Serious | Not formally assessed (<10 estimates) | Single-study evidence | Very low | One study found an association, but replication is required. |
| Prevalent DKD | Observational cross-sectional | 6 articles; 6 categorical estimates | Cross-sectional T2DM samples | OR 2.03 (95% CI 1.71 to 2.42); PI 1.68 to 2.46 | Serious | Not Serious | Serious | Not Serious | Not formally assessed (<10 estimates) | Cross-sectional design precludes assessment of temporality and permits reverse causation | Very low | Higher RC was associated with prevalent or early DKD, but temporality and causality cannot be established from cross-sectional evidence. |

GRADE, Grading of Recommendations Assessment, Development and Evaluation; RC, remnant cholesterol; DKD, diabetic kidney disease; ESKD, end-stage kidney disease; PI, prediction interval. The exploratory mixed-outcome overview models were not graded as uniform causal effects.

# **Table S4.** Continuous and dose-response analyses for incident DKD

| **Analysis or cohort** | **Data and contrast** | **Estimate** | **95% CI** | **P value** | **Heterogeneity or model** | **Interpretation** |
| --- | --- | --- | --- | --- | --- | --- |
| Chen 2025-1 | Directly reported continuous HR, rescaled from 1.0 to 0.1 mmol/L | 1.04 | 1.02-1.06 | <0.001 | Source multivariable Cox model | Study-specific continuous estimate |
| Chen 2025-2 | Directly reported continuous HR, rescaled from 1.0 to 0.1 mmol/L | 1.01 | 1.00-1.03 | 0.068 | Source multivariable Cox model | Study-specific continuous estimate |
| Dai 2025 | Directly reported continuous HR, rescaled from 1.0 to 0.1 mmol/L | 1.01 | 0.99-1.02 | 0.364 | Source multivariable Cox model | Study-specific continuous estimate |
| **Pooled continuous model** | 3 cohorts from 2 articles; per 0.1 mmol/L | 1.02 | 0.98-1.07 | 0.197 | I2=81.4%; tau2=0.000251; Q=9.85 (P=0.007); PI 0.94-1.11 | Direct continuous evidence; common effect not statistically significant |
| Primary assigned doses | 5 cohorts; 13 non-reference category HRs | 1.04 | 1.02-1.06 | 0.008 | One-stage REML meta-regression with study-cluster robust variance | Assumption-dependent linear sensitivity estimate |
| Alternative open-ended doses | 5 cohorts; 13 non-reference category HRs | 1.02 | 1.00-1.05 | 0.030 | One-stage REML meta-regression with study-cluster robust variance | Assumption-dependent linear sensitivity estimate |
| Primary assigned doses excluding Jang 2024 | 4 cohorts; 11 non-reference category HRs | 1.05 | 1.04-1.07 | 0.002 | One-stage REML meta-regression with study-cluster robust variance | Assumption-dependent linear sensitivity estimate |
| **Primary restricted cubic spline** | 3 knots: 0.250, 0.645, and 0.932 mmol/L | NA | NA | Overall, 0.013; nonlinear 0.340 | One-stage multilevel REML spline with study-cluster robust variance | Overall positive association; no significant pooled nonlinearity or common inflection point |
| Model-predicted HR at RC 0.60 mmol/L | Reference RC 0.40 mmol/L | 1.10 | 1.02-1.19 | NA | Primary assigned-dose spline | Exploratory prediction; not a clinical threshold |
| Model-predicted HR at RC 0.80 mmol/L | Reference RC 0.40 mmol/L | 1.19 | 1.08-1.30 | NA | Primary assigned-dose spline | Exploratory prediction; not a clinical threshold |
| Model-predicted HR at RC 1.00 mmol/L | Reference RC 0.40 mmol/L | 1.26 | 1.14-1.39 | NA | Primary assigned-dose spline | Exploratory prediction; not a clinical threshold |

RC, remnant cholesterol; DKD, diabetic kidney disease; HR, hazard ratio; PI, prediction interval; REML, restricted maximum likelihood. For category-based analyses, reported means or medians were used when available; otherwise, interval midpoints and prespecified open-end extensions were assigned. Values in mg/dL were converted using 38.67 mg/dL per mmol/L. The reconstructed dose-response analyses were post hoc and should not be interpreted as defining a treatment target or universal threshold. Chen 2025-1 denotes the UK Biobank cohort and Chen 2025-2 denotes the Chinese Diabetes Registry cohort.
